# Supplementary material for: How Have Researchers Acknowledged and Controlled for Academic Work Activity When Measuring Medical Students’ Internet Addiction? A Systematic Literature Review
Source: Int J Environ Res Public Health. 2021 Jul 20;18(14):7681. doi: 10.3390/ijerph18147681 (PMC8306379; doi:10.3390/ijerph18147681)
Supplement: Supplementary file 1 [file ijerph-18-07681-s001.zip › TableS1.pdf]

Table S1: General Description of Studies

Lead Author, Publication Date, Citation	Year of study	Sample country	Sample Size and Description	Study method (if stated).	Tools or methods used to measure addiction
Ade, 2018 [24]	n.d.	n.p.	N=100 undergraduate medical students (50 female and 50 male) in a "government medical college in a metro city"	Cross sectional, descriptive	Young's IAT (20 Question)
Agah, 2016 [25]	2015	Iran	N=1,000 medical students from Sabzevar University	Cross sectional, descriptive	Young's IAT (20 Question)
Ahmed, 2019 [26]	2018	Bangladesh	N=350 medical students from private medical colleges in Dhaka City	Cross sectional	Young's IAT (20 Question)
Ahmer, 2018 [27]	2016	Pakistan	N=340 medical students from Jinnah Sindh Medical University & Liaquat College of Medicine and Dentistry	Cross sectional	Young's IAT (20 Question)
Alfadhul, 2018 [28]	2017	Iraq	N=218 medical students of faculty of medicine, Kufa University	Systematic sampling; cross sectional	Young's IAT (20 Question)
Ali, 2019 [29]	n.d.	Kashmir	N=100 medical students in Mahi-ud-din Islamic Medical college Mirpur, Kashmir	Convenience sampling; Cross-sectional	Young's IAT (20 Question)

Lead Author, Publication Date, Citation	Year of study	Sample country	Sample Size and Description	Study method (if stated).	Tools or methods used to measure addiction
Alpaslan, 2015 [30]	2013	Turkey	N=328 medical students at Afyon Kocatepe University	Cross-sectional	Young's IAT (20 Question?)
Al-qadasi, 2018 [8]	2015/2016	Yemen	N=275 medical students at University of Science and Technology, Sana'a	Cross sectional	Young's IAT (20 Question)
Anand, 2018 [31]	n.d.	India	N=1763 medical students from three south Indian cities of Bangalore, Mangalore and Trissur	Cross sectional	Young's IAT (20 Question)
Arya, 2018 [32]	2017	India	N=402 MBBS students at medical college, Jhansi, Uttar Pradesh	Cross sectional	Young's IAT (20 Question)
Asokan, 2019 [33]	2017/2018	India	N=381 in "a medical college" in India	Cross sectional	Young's IAT (20 Question)
Bakarman, 2017 [34]	2013/2014	Saudi Arabia	N=161 senior medical students at King Abdulaziz University	Stratified sampling; cross sectional	Young's IAT (20 Question)
Balhara, 2015 [35]	n.d.	Croatia, India, Nigeria	N=842 Graduate medical students at three institutions		Young's IAT (20 Question)
Bansode, 2019 [36]	2018	India	N=83 1st-year medical students, Mumbai	Cross sectional	Young's IAT (20 Question)
Berner, 2014 [37]	2012	Chile	N=384 1st-5th-year undergraduate medical students at a medical		Young's IAT (20 Question)

Lead Author, Publication Date, Citation	Year of study	Sample country	Sample Size and Description	Study method (if stated).	Tools or methods used to measure addiction
			school in Santiago de Chile		
Boonvisudhi, 2017 [38]	2015	Thailand	N=705 1st-5th-year medical students studying at the Faculty of Medicine, Ramathibodi Hospital	Cross-sectional, descriptive	Young's IAT (8 Question)
Capetillo-Ventura, 2015 [39]	2013	n.p.	N=522 medical students		Young's IAT (20 Question)
Chakraborti, 2016 [40]	n.d.	India	N=98 medical undergraduates		Young's IAT (20 Question)
Chathoth, 2013 [41]<br><br>Chathoth, 2014 [42]	2013	India	N=90 medical students	Random sampling; Cross-sectional	Young's IAT (20 Question)
Chaudhari, 2015 [43]	n.d.	India	N=282 medical students	Stratified sample; cross-sectional study	Young's IAT (20 Question)
Chaudhuri, 2019 [44]	n.d.	India	N=201 undergraduate students in a tertiary medical college of West Bengal	Cross-sectional	Young's IAT (20 Question)
Ching, 2017 [45]<br><br>Mooi, 2019 [46]	2013	Malaysia	N=426 medical students from Universiti Putra	Cross-sectional	Young's IAT (20 Question) (Malay Version).
Damor, 2018 [47]	n.d.	India	N=313 medical students of	Cross-sectional	Young's IAT (20 Question)

**Lead Author, Publication Date, Citation**	**Year of study**	**Sample country**	**Sample Size and Description**	**Study method (if stated).**	**Tools or methods used to measure addiction**
			Government Medical College, Surat		
Daripelly, 2017 [48]	n.d.	India	N=150 undergraduate students of final year MBBS, SVS Medical College, Mahabubnagar	Cross-sectional	Young's IAT (20 Question)
Duică, 2017 [49]	n.d.	Romania	N=1276 students from all Romanian medicine universities		Young's IAT (20 Question)
Fatehi, 2016 [50]	2013/2014	Iran	N=174 4th - 7th-year undergraduate medical students at Tehran University of Medical Sciences	Cross-sectional	Young's IAT (20 Question)
Gaddala, 2017 [51]	2017	India	N=402 2nd - 4th year medical students, Chalmeda Anand Rao Institute of Medical Sciences	Random sample; Cross sectional	Young's IAT (20 Question)
Gedam, 2016 [52]	2015	India	N=249[a] 3rd & 4th-year medical students of Jawaharlal Nehru Medical College	Cross-sectional	Young's IAT (20 Question)
Gedam, 2016 [53]	2015	India	N=390 medical students from Wardha, Maharashtra	Random sampling; Cross-sectional	Young's IAT (20 Question)

**Lead Author, Publication Date, Citation**	**Year of study**	**Sample country**	**Sample Size and Description**	**Study method (if stated).**	**Tools or methods used to measure addiction**
George, 2019 [9]	n.d.	India	N=199[b] medical students "in a private medical college"	Stratified, randomised; Cross-sectional	Young's IAT (20 Question)
Ghamari, 2011 [54]	2009	Iran	N=426 medical students of Arak University	Stratified proportional sampling; random sampling	"Yang" [Young's IAT?] Persian language version.
Ghanate, 2019 [55]	2018	India	N=700 medical students of Kalaburagi district of Karnataka	Random sampling ; cross-sectional	Young's IAT (20 Question)
Ghosh, 2018 [56]	2015	India	N= 155 1st and 3rd semester medical students of CMSDH, Kamarhati, West Bengal	Cross-sectional	Young's IAT (20 Question)
Güzel, 2018 [57]	2017/2018	Turkey	N=327 medical students of Trakya University School of Medicine	Stratified, randomised sampling	Günüç Murat Kayri's Internet Addiction Scale (Günüç & Kayrı, 2010)
Hajare, 2017 [58]	2014/2015	India	N=402 medical students at NKP Salve Institute of Medical Sciences & Research Centre, Nagpur	Cross-sectional; Stratified random sampling	Young's IAT (no. of questions not given)
Hamissi, 2013 [59]	n.d.	n.p.	N=201 medical students	Cross–sectional survey;	Young's IAT (20 Question)

**Lead Author, Publication Date, Citation**	**Year of study**	**Sample country**	**Sample Size and Description**	**Study method (if stated).**	**Tools or methods used to measure addiction**
				random selection	
Haque, 2016 [60]	2014/2015	Malaysia	N=112 1st-5th year medical students of Universiti Sultan Zainal Abidin	Random selection; cross-sectional	Internet Addiction Diagnostic Questionnaire (http://netaddiction.com/internet-addiction-test/)
Hayat, 2020 [61]	n.d.	Iran	N=233 medical students of Shiraz University of Medical Sciences	Cross-sectional correlational	Young's IAT (20 Question)
Hussain, 2018 [62]	2015	Pakistan	N=120 medical students	Random Sampling	Young's IAT (20 Question)
Ja'ffar, 2019 [63]	2017	Iraq	N=263 Al-Kindy Medical College Students in Baghdad	Cross sectional	Young's IAT (20 Question)
Jain, 2018 [64]	2017	India	N=150 undergraduate medical students of SS Medical College Rewa, MP	Randomised; Cross sectional	Young's IAT (20 Question)
Javaeed, 2019 [65]	2018	Kashmir (Pakistan)	N=210 undergraduate medical students (first to the fifth year) Poonch Medical College, Azad Kashmir	Cross-sectional	Young's IAT (20 Question)
Javaeed, 2020) [66]	2018	Kashmir (Pakistan)	N=316 medical students (second to fifth year) of Poonch Medical College, Azad Kashmir	Cross-sectional	Young's IAT (20 Question)

**Lead Author, Publication Date, Citation**	**Year of study**	**Sample country**	**Sample Size and Description**	**Study method (if stated).**	**Tools or methods used to measure addiction**
Kannan, 2019 [67]	2016	India	N=201 undergraduate medical students	Cross-sectional	Young's IAT (20 Question)
Kapadia, 2015 [68]	2015	India	N=200 medical students of B. J. Medical College, Ahmedabad	Random selection	Young's IAT (20 Question)
Khan, 2016 [69] Khan, 2017 [70]	2015	Pakistan	N=322 MBBS students at Army Medical College, Rawalpindi	Non-probability convenience sampling	Young's IAT (20 Question)
Komleh, 2015 [71]	2013	Iran	N=417 medical students of basic sciences of Tehran University of Medical Sciences	Stratified random sampling; cross-sectional	Young's IAT (no. of questions not given) [Farsi version]
Kootesh, 2016 [10]	n.d.	Iran	N= 250 Tehran University of medical sciences University students	Stratified Random sample	Young's IAT (20 Question)
Kumar, 2017 [72]	2015	India	N=150[c] medical students of SSIMS and RC Davangere	Random sampling; cross sectional study	Young's IAT (20 Question)
Kundu, 2017 [73]	2016/2017	India	N=130 medical students of Gurugram	Cross-sectional	Young's IAT (20 Question)
Latt, 2017 [74]	2015	Malaysia	N=103 first year medical students at	Cross-sectional descriptive	Young's IAT (20 Question)

**Lead Author, Publication Date, Citation**	**Year of study**	**Sample country**	**Sample Size and Description**	**Study method (if stated).**	**Tools or methods used to measure addiction**
			IIUM, Kuantan Campus, Pahang		
Liu, 2009 [75]	2009	China	N=352 students from three medical academies in Xi'an	Randomly stratified cluster sampling	Author's own "Questionnaire on University Students' Internet Behavior"
Loredo e Silva, 2018 [76]	2015/2016	Brazil	N=710 students from Federal University of Juiz de Fora	Cross-sectional	Young's IAT (20 Question)
Madhusudan, 2018 [77]	2018	India	N=729 medical students at DM Wayanad Institute of Medical Sciences, Wayanad District, Kerala	Cross-sectional	Young's IAT (20 Question)
Mallya, 2019 [78]	2018	n.p.	N=200 1st & 2nd-year medical students at a private medical college	Cross-sectional	Chen's Internet Addiction Scale (CIAS)
Malviya, 2014 [79]	2012	India	N=242 undergraduates of MGM Medical College of Indore City	Random sampling; cross sectional	Young's IAT (20 Question)
Mohammadbeigi, 2016 [80]	2014	Iran	N=254 students in Qom Medical University, second term or higher	Cross sectional	Young's IAT (20 Question)
Mostafa, 2019 [81]	2017/2018	Bangladesh	N=254[a] medical students from Chattagram Maa-O-Shishu Hospital	Cross-sectional	Young's IAT (20 Question)

**Lead Author, Publication Date, Citation**	**Year of study**	**Sample country**	**Sample Size and Description**	**Study method (if stated).**	**Tools or methods used to measure addiction**
			Medical College (CMOSHMC)		
Mukherjee, 2020 [82]	2015	India	N=150 medical students, aged 17-25, in R.G Kar Medical College, Kolkata	Cross-sectional	Young's IAT (20 Question)
Nagori, 2016 [83]	2014/2015	India	N=525 Medical students [from Government Medical College, Bhavnagar?]	Observational, cross-sectional	Young's IAT (20 Question)
Nath, 2016 [84]	2015	India	N=188 medical students from Silchar Medical College and Hospital	Cross-sectional	Young's IAT (20 Question)
Öztürk Kaygusuz, 2019 [85]	2018	Turkey	N=407 Medical Faculty students of Fırat University	Cross-sectional	Modified Young's IAT (20 Question) [Turkish version]
Patel, 2018 [86]	2018	India	N=139 2nd-year MBBS students	Cross-sectional	Young's IAT (20 Question)
Patil, 2017 [87]	2016	India	N=488 medical students in Nagpur	Cross-sectional	Young's IAT (20 Question)
Pramanik, 2012 [88]	2012	Nepal	N=130 medical students	Random sampling; cross-sectional	Young's IAT (20 Question)
Qadir, 2018 [89]	2018	n.p.	N=100 medical students from Gomal Medical College	Cross-sectional	Young's IAT (20 Question)
Radeef, 2018 [90]	n.d.	Malaysia	N=268 medical students from	Prospective, cross-sectional	Chen Internet addiction Scale (Malay version)

Lead Author, Publication Date, Citation	Year of study	Sample country	Sample Size and Description	Study method (if stated).	Tools or methods used to measure addiction
			International Islamic University Malaysia, Kuantan campus		
Rebello, 2017 [91]	2016	India	N=129 1st-year medical students Karwar Institute of Medical Sciences, Karwar, Karnataka	Cross-sectional	Compulsive internet usage scale (Meerkerk, Van Den Eijnden, Vermulst, & Garretsen, 2009)
Rustam, 2017 [92]	2016	Pakistan	N=200 medical students from Rehman Medical College, Peshawar	Cross-sectional	Young's IAT (20 Question)
Sahraian, 2016 [93]	2014/2015[d]	Iran	N=278 medical students	Cross-sectional	Young's IAT (20 Question)
Saied, 2016) [94]	2015	Egypt	N=2,200 Malaysian and Egyptian medical students in Tanta faculty of medicine, Tanta University	Cross-sectional	Young's IAT (20 Question)
Saini, 2016 [95]	2015	India	N=140 medical students from the medical college of Bikaner	Cross-sectional	Young's IAT (20 Question)
Salehi, 2014 [96]	2013	Iran	N=383 medical students from Mashad, Iran	Cross sectional	Chen Internet addiction Scale (Farsi version)
Salek Ebrahimi, 2019 [97]	2018	Iran	N=96 medical students of Shahid Beheshti	Random sampling,	Young's IAT (20 Question)

Lead Author, Publication Date, Citation	Year of study	Sample country	Sample Size and Description	Study method (if stated).	Tools or methods used to measure addiction
			University of Medical Sciences	cross-sectional	
Samaha, 2019 [98] Samaha, 2018 [99]	2017	Lebanon	N=596 medical students in "various medical schools in Beirut"	Correlational, cross-sectional	Young's IAT (20 Question)
Şenol, 2019 [100]	n.d.	Turkey	N=168 1st-3rd year medical students from Suleyman Demirel University		(Günüç & Kayrı, 2010)
Shadzi, 2020 [101]	2018	Iran	N=402 1st-7th year medical students of Shiraz University of Medical Sciences	Stratified, randomised, cross-sectional	Young's IAT (20 Question) [Persian Version]
Shi, 2019 [102]	2017	China	N=1,264 medical students from China Medical University, Guizhou Medical University, and Xiangya School of Medicine	Cross-sectional	Young's IAT (20 Question)
Shinde, 2018 [103]	2017/2018	India?	N=90 students of 1st-3rd year "in government medical college"	Randomised, cross sectional	Young's IAT (20 Question)
Shoghli, 2018) [6]	2018	Iran	N= 137 internship students Zanjan University of Medical Sciences	Cross-sectional	Young's IAT [20 Question?] [Persian version]

**Lead Author, Publication Date, Citation**	**Year of study**	**Sample country**	**Sample Size and Description**	**Study method (if stated).**	**Tools or methods used to measure addiction**
Simcharoen, 2018 [104]	2015	Thailand	N= 324 1st-6th-year medical students of Chiang Mai University		Young's IAT (20 Question)
Singh, 2018 [105]	n.d.	India	N=149 undergraduate students at Sikkim Manipal Medical College		Young's IAT (20 Question)
Singh, 2018 [106]	2017	India	N=122 medical undergraduates in "a medical college in Northern India"	Cross-sectional	Young's IAT (20 Question)
Siraj, 2015 [107]	2011/2012	Malaysia	N=176[e] 4th-year medical students at Malaysian Public University	Cross-sectional	Young's IAT (8 Question)
Srijampana, 2014 [108]	2013	India	N=211 medical students from two medical colleges in Guntur, Andhra Pradesh	Cross-sectional	Young's IAT (20 Question)
Subhaprada, 2017 [109]	2016	India	N=95 undergraduate students of II MBBS, in Kurnool Medical College, Kurnool, Andhra Pradesh	Simple random sampling; cross-sectional	Young's IAT (20 Question)
Suresh, 2018 [110]	n.d.	India	N=150 1st-year medical students of Akash Institute of Medical	Cross-sectional	Young's IAT (20 Question)

Lead Author, Publication Date, Citation	Year of study	Sample country	Sample Size and Description	Study method (if stated).	Tools or methods used to measure addiction
			Sciences and Research Centre, Bangalore		
Sushma, 2018 [111]	2015	India	N= 236 undergraduate medical students in Mysore Medical College and Research Institute, Mysuru	Cross-sectional	Young's IAT (20 Question)
Taha, 2019 [11]	2017/2018	Saudi Arabia	N=209[f] 1st-3rd year medical students in Qassim University	Cross-sectional	[Young's?] IAT [own Arabic translation] (20 Question)
Tan, 2019 [7]	n.d.	Malaysia	N=207 medical students in Universiti Putra Malaysia	Cross-sectional	Young's IAT (8 Question)
Tsimtsiou, 2014 [112]	2012	Greece	N=140 undergraduate and postgraduate medical students		Young's IAT (20 Question) [Greek version]
Tsimtsiou, 2015 [113]	2013	Greece	N=585 medical students at the Aristotle University of Thessaloniki School of Medicine		Young's IAT (20 Question) [Greek version]
Upadhayay, 2017 [114]	n.d.	Nepal	N=100 1st and 2nd year medical students at Gandaki Medical College, Lekhnath	Convenience sample; cross-sectional	Young's IAT (20 Question)
V, 2017 [115]	2015/2016	India	N=350 medical students from Mamata Medical College, Khammam	Random sampling; cross-sectional	Young's IAT (20 Question)

Lead Author, Publication Date, Citation	Year of study	Sample country	Sample Size and Description	Study method (if stated).	Tools or methods used to measure addiction
Vidyachathoth, 2013 [116]	n.d.	India	N=90 medical students from Mangalore.	Randomised, cross-sectional	Young's IAT (20 Question)
Wang, 2020 [117]	2018	China	N=3,738 medical students from Wannan Medical College, China	Stratified per year, randomised	Young's IAT (20 Question)
Yerpude, 2019 [118]	2016	India	N=312 final year MBBS students of a private medical college in Gurat	Cross-sectional	[Young's?] IAT (20 Question)
Yücens, 2018 [119]	2017/2018	Turkey	N=392 undergraduate medical students at Afyon Kocatepe University	Cross-sectional	Young's IAT (20 Question)

[a] Part of a larger study.
[b] Not given in the paper, but estimated based upon percentages.
[c] Abstract says 138, but paper shows 150.
[d] Dates given as 1393-1394 (Persian Calendar)
[e] Abstract says 186, but data reflects only 176.
[f] N given as 216, but only 209 completed the survey.

Table S2: Detailed Results

Lead Author, Publication Date, Citation	Validity of IA questioned?	Measured against (e.g. Depression	Age & Gender	Addiction rates (Prevalence)	Mentioned value of Internet for Academic work	Results
Ade, 2018 [24]	No	None		None: 11%<br>Mild: 63%<br>Moderate: 20%<br>Severe: 6%	Yes.<br>Acknowledged in Introduction only, then no further discussion.	No usage or correlations studied.
Agah, 2016 [25]	No	None	Mean age: 21.1±3.5	None: 36%<br>Mild: 51.1%<br>Moderate: 12.3%<br>Severe: 0.6%	Yes.<br>Acknowledged in Introduction; single mention in Discussion, but not taken into account	IA correlated with:<br>• gender (M>F)<br>• marital status<br>• indigenous status.
Ahmed, 2019 [26]	No	None	Mean age: 21.1±1.5<br>Female: 73.7%<br>Male: 26.3%	None: 23.1%<br>Mild: 17.8%<br>Moderate: 48.1%<br>Severe: 11.0%	Acknowledged in Introduction.<br><br>Mentioned in Discussion, but not taken into account.	Internet used for:<br>• entertainment: 43.7%<br>• time passing: 40%<br>• social networking: 9.7%<br>• *academic: 6.6 %*<br><br>IA correlated with:<br>• academic problems<br>• time spent on the Internet.
Ahmer, 2018 [27]	No	None	Age: 21.20 ±1.67	None: 15%<br>Mild: 65.6%<br>Moderate: 18.5%<br>Severe: 0.9%	No.<br>No mention in Introduction. Academic results ignored in Discussion.	39.7% had IA and ignored academic work;<br>44.5% had AI and did not ignore academic work.

Lead Author, Publication Date, Citation	Validity of IA questioned?	Measured against (e.g. Depression	Age & Gender	Addiction rates (Prevalence)	Mentioned value of Internet for Academic work	Results
						IA correlated with:<br>• gender (F>M)
Alfadhul, 2018 [28]	No	None	Mean age 19.29±1.04<br>Female: 50.9%<br>Male: 49.1%	None-Mild: 44.5%<br>Moderate: 54.6%<br>Severe: 0.9%	Yes. Acknowledged in Introduction. Measured percentage of work-related time on Internet. Discussion does not discuss significance of the work-related figures on the Addiction rates.	Percentage of work-related time on Internet:<br>Normal: 67.7%<br>Addicted: 32.8%<br><br>No correlation between IA and academic performance
Ali, 2019 [29]	No	None	Mean age: 19.71±0.90	None-Mild: 46%%<br>Moderate: 53%<br>Severe: 1%<br>Males more than females.	Yes. Acknowledged in Introduction.<br>Measured academic use, and mentioned in the Discussion, but did not question the validity of the addiction rate.	54% of students used it for work-related purposes
Alpaslan, 2015 [30]	No	Hopelessness, Suicide Ideation and Hostility	Mean age: 20.5±1.8<br>Female: 55.8%<br>Male: 44.2%	None: 70.4%[a]<br>Mild: 23.3%<br>Moderate: 5.5%<br>Severe: 0.9%	No.<br><br>The correlation between AI and higher grades found in the study dismissed "probably	Positive relationship between problematic internet use and Hopelessness, Suicide Ideation and Hostility.<br><br>IA correlated with:<br>• daily use

**Lead Author, Publication Date, Citation**	**Validity of IA questioned?**	**Measured against (e.g. Depression**	**Age & Gender**	**Addiction rates (Prevalence)**	**Mentioned value of Internet for Academic work**	**Results**
		UCLA loneliness scale; Toronto Alexithymia Scale (TAS); Suicide Probability Scale (SPS)			related to self-report of the students"	• years of use<br>• gender (M>F)<br>• game playing<br>• UCLA<br>• TAS<br>• SPS<br>• higher grades
Al-qadasi, 2018 [8]	No (States that it is in DSM-IV)	None	Mean age: 21.6±1.98. Female: 49.8% Male: 50.2%	None: 18.5%<br>Mild: 68.7%<br>Moderate: 12.4%<br>Severe: 0.4%	Yes, acknowledged in Introduction. And not examined or discussed.	IA correlated with:<br>• more than 10 friends<br>• time on Internet<br>• higher Internet speeds
Anand, 2018 [31]	No	Psychological distress and depression	Mean age: 19.73±1.35	None: 61.8%[a]<br>Mild: 27%<br>Moderate: 10.4%<br>Severe: 0.8%	No. No mention of academic work.<br><br>The high % use of the Internet for work ignored in the Discussion.	Internet used for:<br>• *education: 8.3%*<br>• entertainment: 6.8%<br>• messaging: 2.9%<br>• *all: 81.9%*<br><br>IA correlated with:<br>• gender (M>F)<br>• amount of Internet usage

Lead Author, Publication Date, Citation	Validity of IA questioned?	Measured against (e.g. Depression	Age & Gender	Addiction rates (Prevalence)	Mentioned value of Internet for Academic work	Results
						• psychological distress<br>• inverse with age<br><br>Some balanced discussion of possible causation between psychological distress and IA.
Arya, 2018 [32]	No	None	Female: 41.55%<br>Male: 58.45%	None-Mild: 90.54%<br>Moderate: 6.21%<br>Severe: 3.23%	No. No discussion of work/education related activities.	IA correlated with:<br>• gender (M>F)<br>• rural background > urban background<br>• Internet access at home > than those without.
Asokan, 2019 [33]	No (although acknowledges not in the DSM)	None	Female: 71.9%<br>Male: 28.1%	None: 38.6%[a]<br>Mild: 63.7%<br>Moderate: 35.5%<br>Severe: 0.8%	Yes, Acknowledged in the Introduction. Value in medical practice also noted.<br><br>Mentions its academic use briefly, but does not lead to any insights about the value of "addiction".	Usage:<br>• 12.5% who use it for academic work were in the non-addicted group,<br>• 87.5% were in the addicted group.<br><br>IA correlated with:<br>• gender (M>F)<br>• time spent on Internet<br>• negative with grades

**Lead Author, Publication Date, Citation**	**Validity of IA questioned?**	**Measured against (e.g. Depression**	**Age & Gender**	**Addiction rates (Prevalence)**	**Mentioned value of Internet for Academic work**	**Results**
Bakarman, 2017 [34]	No	Depression (Center for epidemiological studies depression scale for children (CES-DC))	Mean age: 22.2±1.0<br>Female: 57.8%<br>Male: 42.2%[a]	IA[b]:<br>None: 50.6%[a]<br>Possible: 46.3%<br>Addicted: 3.1%	Acknowledged in the Introduction.<br><br>Impact not discussed elsewhere.	IA correlated with:<br>• gender (M > F)<br>• level of Depression<br>• inverse with year of study.
Balhara, 2015 [35]	No, (although acknowledges that it is not in the DSM)	None	Mean age: 21.23±2.66<br>Female: 50.8%<br>Male: 49.2%	None: 50.3% [a]<br>Mild: 38.7%<br>Moderate: 10.5%<br>Severe: 0.5%	No. No discussion of work-related activities.	IA correlated with:<br>• gender (M>F)<br>• average duration of daily use of the internet<br>• social networking<br>• chatting<br>• gaming<br>• shopping<br>• viewing pornography
Bansode, 2019 [36]	Yes - In the conclusion,	None	Female: 43.4%<br>Male: 56.6%	Less than average user: 6.87%<br>Average user: 67.47	Not in Introduction.<br><br>Their high education figures are referred to, but	Internet used for:<br>• social networking: 73.49%<br>• *education: 68.87%*

Lead Author, Publication Date, Citation	Validity of IA questioned?	Measured against (e.g. Depression	Age & Gender	Addiction rates (Prevalence)	Mentioned value of Internet for Academic work	Results
	mention that it is debated and not in DSM.			Over-users: 12.05<br>Addicts: 6.38	do not impact on their interpretation of IA.	• downloading: 62.65%<br>• gaming: 12.05%<br><br>IA correlated with:<br>• gender (M>F)
Berner, 2014) [37]	Yes, acknowledges that IA is not firmly established	Depression (Goldberg's General Health Questionnaire (GHQ-12))	Mean age: 20.78±1.79<br>Male: 58.3%	IAT:<br>Non-problematic (<50 on score):88.3%[a]<br>Problematic (>=50 on score):11.7%	No.<br><br>No mention of academic work in the paper.	IA correlated with:<br>• GHQ-12
Boonvisudhi, 2017 [38]	No, although mentions that there is no consensus on the clinical definition.	Depression (The Patient Health Questionnaire (PHQ-9))		Mean IA score = 3.27;<br><br>24.4% had possible IA.	No.	IA correlated with:<br>• depression<br>• academic problems<br>• health problems

Lead Author, Publication Date, Citation	Validity of IA questioned?	Measured against (e.g. Depression	Age & Gender	Addiction rates (Prevalence)	Mentioned value of Internet for Academic work	Results
Capetillo-Ventura, 2015 [39]	No	Mental Health General Health Questionnaire, and the Zuckerman-Kuhlman Personality Questionnaire III (ZKPQ)	Mean age: 21.24±3.046<br>Female: 46.2%<br>Male: 53.8%	IAT<br>Mean score: 19.72<br><br>IA:<br>had complete control over its use (20-39): 91.8%<br>frequent problems: (40-69): 8%<br>significant problems: (>70): 0.2%.	Yes<br>Acknowledged in Introduction.<br><br>The extremely high use for education and research ignored in the discussion. It is mentioned in the conclusion, but does not impact of the interpretation of the results.	Internet main usage for:<br>• social networks: 43.86%<br>• *academic and research activities: 32.95%*<br>• entertainment/leisure in 23.18%.<br><br>IA correlated with:<br>• gender (M>F)
Chakraborti, 2016 [40]	No	Resilience scale and student stress scale (SSS).	Mean age: 20.41±1.64<br>Female: 35.7%<br>Male: 64.3%	IA:<br>Moderate users: 80.6%<br><br>19.4% were problem users.	No.<br><br>Mentioned in the Discussion (that is among the highest uses of the Internet), but not taken into account with the interpretation.	Internet used for:<br>• social networking: 76.5%<br>• *academic activity and research: 74.5%*<br><br>IA correlated with:<br>• gender (M>F)<br>• SSS total score<br>• total number of stressful life events

Lead Author, Publication Date, Citation	Validity of IA questioned?	Measured against (e.g. Depression	Age & Gender	Addiction rates (Prevalence)	Mentioned value of Internet for Academic work	Results
						• negatively correlated with resilience
Chathoth, 2013 [41]<br><br>Chathoth, 2014 [42]	No	None	Mean age: 18.49± 0.71<br><br>Female: 62.2%<br>Male: 37.8%	IA:<br>Normal: 23.33%<br>Mild: 57.77%<br>Moderate: 18.88%<br>Severe: 0%	No<br><br>Brief mention in the Discussion, but not taken into account on the interpretation of the results.	Internet used for:<br>• social networking: 97.8%<br>• e-mail: 87.8%<br>• *education: 82.2%*<br>• entertainment: 82.2%<br><br>IA correlated with:<br>• sleep impairment<br>• excessive daytime sleepiness<br>• environmental stressors
Chaudhari, 2015 [43]	No, but acknowledges not in DSM.	None	Mean age: 19.90±1.37<br>Female: 56.74%<br>Male: 43.26%	IA:<br>None: 41.13%<br>Mild: 51.42%<br>Moderate: 7.45%	Acknowledged in Introduction. Although, give a list of 5 aspects that make students more vulnerable to AI, and education is not mentioned.<br><br>No mention of academic work in the Discussion.	IA correlated with:<br>• gender (M>F)<br>• staying in private accommodation<br>• lesser age of first internet use<br>• using mobile for internet access<br>• higher expenditure on internet<br>• staying online for longer time

**Lead Author, Publication Date, Citation**	**Validity of IA questioned?**	**Measured against (e.g. Depression**	**Age & Gender**	**Addiction rates (Prevalence)**	**Mentioned value of Internet for Academic work**	**Results**
						• using internet for social networking, online videos<br>• watching website with sexual content
Chaudhuri, 2019 [44]	No	The Duke Health Profile		IA:<br>None: 25.87<br>Mild: 58.70<br>Moderate: 15.42	No.<br><br>High usage for educational purposes mentioned in Discussion, but does not affect the interpretation of the IA.<br><br>Recommendations include avoid internet at night, during classes and lectures, in spite of the 83% usage for education.	Internet used for:<br>• *education: 83.0%*<br>• social networking: 76%<br><br>IA correlated with:<br>• Anxiety score<br>• Depression score<br><br>IA negatively correlated with:<br>• Physical health score<br>• Mental health score<br>• Perceived health score
Ching, 2017 [45]<br><br>Mooi, 2019 [46]	No	Depression	Mean age: 21.60±1.50<br>Female: 63.4%<br>Males: 36.6%	Addicted: 36.9%	Yes.<br>Acknowledged in Introduction as important for medical students for “literature searches and searching for relevant medical information.”	*51.6% use Internet for education purposes.*<br><br>IA correlated with:<br>• gender (M>F)<br>• duration of internet use<br>• DASS-21 Score

**Lead Author, Publication Date, Citation**	**Validity of IA questioned?**	**Measured against (e.g. Depression**	**Age & Gender**	**Addiction rates (Prevalence)**	**Mentioned value of Internet for Academic work**	**Results**
					The education usage is not mentioned in the Discussion, and does not influence the interpretation.	
Damor, 2018 [47]	No	None	Female: 48.6%<br>Male: 51.4%	IA:<br>Less than average users: 23.4%<br>Average online users: 59.1%<br>Possible addict: 17.2%<br>Addict: 0.3%	Yes, Acknowledged in Introduction.<br><br>Mentioned in the Discussion, but no impact on the interpretation of results.	Internet used for:<br>• social media: 65.2%<br>• *academic literature search: 19.4%*
Daripelly, 2017 [48]	No	General health questionnaire (GHQ - 12)	Female: 49.4%<br>Male: 50.6%	IA (Medical students only):<br>Non-Addict: 46.3%<br>Potential Addict: 27.5%<br>Internet Addict: 21.6%	No mention in the Introduction.<br><br>The results show that, overall, and for the "potential addicts" the most common use of the Internet is for research purposes. This is ignored in the Discussion and the interpretation of the results.	Internet most commonly used for:<br>• *research: 35.1%*<br>• socialization: 34.7%<br>• entertainment: 24.6%<br>• others: 5.5%<br><br>IA correlated with<br>• gender (M>F)<br>• parents' level of education<br>• more hours of daily internet use

Lead Author, Publication Date, Citation	Validity of IA questioned?	Measured against (e.g. Depression	Age & Gender	Addiction rates (Prevalence)	Mentioned value of Internet for Academic work	Results
						• use the internet more frequently each week<br>• use the internet for socialization and entertainment<br>• GHQ scores
Duică, 2017 [49]	No	None		IA:<br>No/Mild: 91.5%<br>Moderate: 8.2%<br>Severe: 0.4%	Acknowledges some positive contribution of Internet to work-related activities.	Found high use of the Internet for work, and "those with internet addiction are the ones that use the internet as information source for professional development."<br><br>IA negatively correlated with:<br>• watching medical videos before exams<br>• internet socialization (including topics from professional training)
Fatehi, 2016 [50]	No	WHOQOL-BREF	Mean age: 22.57±1.24<br>Female: 55.7% [a]<br>Male: 44.3%	IA:<br>Normal (<50): 83.9%<br>Addicted (>= 50): 16.1%	No.<br><br>No mention of the Mentions Internet as a tool for work.	GPA lower in addicted group.<br><br><br>IA negatively correlated with:

Lead Author, Publication Date, Citation	Validity of IA questioned?	Measured against (e.g. Depression	Age & Gender	Addiction rates (Prevalence)	Mentioned value of Internet for Academic work	Results
						• average of QOL domains
Gaddala, 2017 [51]	No	None	Female: 64%<br>Male: 36%	IA:<br>None: 24%<br>Mild: 61%<br>Moderate: 14%<br>Severe: 1%	Acknowledged in Introduction.<br><br>The fact that 100% of the student use the Internet for educational purposes is ignored in the discussion.	*100% of the students use the Internet for educational purposes.*<br><br>IA correlated with:<br>• Gender (M>F)
Gedam, 2016 [52]	No. Acknowledge the work by [120], but still proceeded.	Depression Mental health inventory	Mean age: 19.71±0.97<br><br>Female: 63.5%<br>Male: 36.5%	IA:<br>None/Mild: 81.5%<br>Moderate: 17.3%<br>Severe: 1.2%	Acknowledged in the Introduction.<br><br>High usage of Internet for educational work is not mentioned in the discussion.	Internet used for:<br>• social networking: 31.86%<br>• *education: 28.92%*<br>• games: 17.91%<br>• recreational: 17.32%<br>• others: 3.96%<br><br>IA correlated with:<br>• gender (M>F)<br>• computer ownership<br>• purpose of internet use
Gedam, 2016 [53]	No	Anxiety, depression, distress	Mean age: 19.57±1.52<br>Female: 62.8%	IA<br>None/Mild: 76.7%<br>Moderate: 21.0%<br>Severe: 2.3%	Yes.<br>Acknowledged in Introduction.<br>High usage of Internet for educational work is not	Internet used for:<br>• social networking: 31.08%<br>• *education: 28.87%*<br>• recreational: 18.26%<br>• games: 16.69%

Lead Author, Publication Date, Citation	Validity of IA questioned?	Measured against (e.g. Depression	Age & Gender	Addiction rates (Prevalence)	Mentioned value of Internet for Academic work	Results
			Males: 37.2%		mentioned in the discussion.	• others: 5.07%<br><br>IA correlated with:<br>• gender: (M>F)<br>• anxiety<br>• depression<br>• loss of emotional / behavioral control<br>• psychological distress<br><br>IA negatively correlated with:<br>• life satisfaction<br>• psychological well-being
George, 2019 [9]	No. And says that the DSM does include internet addiction disorder.	None	Mean age: 21.3±1.25	IA<br>None: 29% [a]<br>Mild: 54%<br>Moderate: 17%	Acknowledged in Introduction.<br><br>Did not ask the students what they did, and so this aspect not covered in the Discussion.	IA correlated with:<br>• gender (M>F)<br>• mobile/tablet usage<br>• eye strain<br>• time to initiate sleep<br>• frequency of sleep trouble<br>• hours spent on internet<br>• money spent<br>• presence of backache<br>• appetite change

Lead Author, Publication Date, Citation	Validity of IA questioned?	Measured against (e.g. Depression	Age & Gender	Addiction rates (Prevalence)	Mentioned value of Internet for Academic work	Results
Ghamari, 2011 [54]	No	None	Mean age: 21[a]<br>Female: 64.9%<br>Male: 35.1%[a]	IA:<br>Mean score: 32.74±14.52<br>Moderate: 8%<br>Severe: 2.8%	Acknowledged in Introduction.<br><br>The fact that the students used the Internet mostly for work is repeated in the Discussion, but does not influence the researchers' interpretation of their view on addiction.	Internet used for:<br>• *research and scientific surveys: 48.4%*<br>• computer games and hobbies: 20.5%<br>• checking email: 9.8%<br>• political and social news: 6.5%<br>• chat rooms: 5.6%<br><br>IA correlated with:<br>• gender (M>F)<br>• marital status (M>S)<br>• father's job<br>• knowledge about computer and internet<br>• educational level.
Ghanate, 2019 [55]	No	Becks Depression scale; Beck's Anxiety Inventory	Female: 53.14%<br>Male: 46.8%	IA:<br>Normal: 80.9%<br>Moderate: 17.4%<br>Severe: 1.7%	Acknowledged in Introduction.<br><br>In spite of these work figures, this is not mentioned in the Discussion, nor does it	Internet used for:<br>• entertainment: 81%<br>• *academic work: 36.1%*<br>• social friendships: 35%<br>• loneliness: 11.4%<br><br>IA correlated with:<br>• Anxiety

Lead Author, Publication Date, Citation	Validity of IA questioned?	Measured against (e.g. Depression	Age & Gender	Addiction rates (Prevalence)	Mentioned value of Internet for Academic work	Results
					impact on the interpretation of the results	• Depression
Ghosh, 2018 [56]	No	None	Female: 44.5%<br>Male: 55.5%	IA:<br>None: 44.5%<br>Mild: 34.8%<br>Moderate: 19.4%<br>Severe: 1.3%	Mentioned in the Abstract, but not in the Paper's Introduction.<br><br>Discussion talks about the importance of medical persons using the Internet to stay up to date, does not affect the interpretation of these results, and does not addressed the anomaly that students appear to not access their learning materials online.	Internet used for:<br>• online chatting: 78.7%<br>• social networking: 61.9%<br><br>IA correlated with:<br>• gender (M>F)<br>• duration of daily usage<br>• amount of money spent for Internet usage

**Lead Author, Publication Date, Citation**	**Validity of IA questioned?**	**Measured against (e.g. Depression**	**Age & Gender**	**Addiction rates (Prevalence)**	**Mentioned value of Internet for Academic work**	**Results**
Güzel, 2018 [57]	No	None	Age range from 18-26.<br>Female: 54.13%<br>Male: 45.87%	[Did not use Young's scale]	Acknowledged in Introduction.<br><br>In spite of the high usage for educational work, no reference is made in the Discussion, and it does not affect their interpretation.	Internet used for:<br>• communicational purposes: 94.5%<br>• *educational purposes: 68.9%*<br><br>IA correlated with:<br>• some physical complaints<br>• insomnia<br>• time spent on the internet
Hajare, 2017 [58]	No	None	Age 21.2[a]<br><br>Female: 42.54%<br>Male: 57.4%	IA:<br>None: 33.58%<br>Mild: 23.38%<br>Moderate: 39.55%<br>Severe: 3.48%	No mention in Introduction, or anywhere else in the paper.	IA correlated with:<br>• both the parents working<br>• had internet connections at their home or hostel room<br>• using internet for more than 5 years<br>• used internet for more than 28 hrs/week<br><br>No correlation with age or gender was found.

**Lead Author, Publication Date, Citation**	**Validity of IA questioned?**	**Measured against (e.g. Depression**	**Age & Gender**	**Addiction rates (Prevalence)**	**Mentioned value of Internet for Academic work**	**Results**
Hamissi, 2013 [59]	No	Emotional Intelligence	Mean age: 23.26±3.36<br>Female: 31.8%[a]<br>Male: 68.2%	IA:<br>Normal: 38.3%<br>Mild: 43.8%<br>Moderate: 15.9%<br>Severe: 2%	No<br><br>No mention of education influence at all, in spite of the high usage of the Internet for Education, and in spite of the fact that the PhD students use the Internet more.	Internet used for:<br>• *read articles: 33.8%*<br>• download music/images: 7.7%<br>• chat: 1.2%<br>• games: 1.5%<br>• not at all: 1%<br><br>Usage of Computer:<br>• games: 43.8%<br>• *education: 25.0%*<br>• communication: 15.4%<br>• entertainment: 5.1%<br>• not at all: 1%<br><br>IA correlated with:<br>• gender (M>F)<br>• education grade: PhD having the highest.<br>• Internet usage<br>• history of depression
Haque, 2016 [60]	No	None	Mean age: 21.99±1.535	Both male and female suffered from mild IA	Acknowledged in Introduction.	Internet used for:<br>• social networking: 56.2%<br>• entertainment: 39.7%

Lead Author, Publication Date, Citation	Validity of IA questioned?	Measured against (e.g. Depression	Age & Gender	Addiction rates (Prevalence)	Mentioned value of Internet for Academic work	Results
			IA Mean Scores:<br>Female: 41.4±13.05<br>Males: 44.9±14.05		Work usage in Results ignored in the Discussion. No reasons given.	• *educational: 34.9%*<br>• *research: 12.3%*<br>• email: 12.3%<br>• conferences: 3%<br><br>IA negative correlated with:<br>• year of study<br>• age<br><br>IA did not correlate with:<br>• usage<br>• work<br>• gender
Hayat, 2020 [61]	No	Procrastination	Female: 57.1%<br>Male: 42.9%	IA:<br>Severe: 3.43%	Yes	IA correlated with:<br>• gender (M>F)<br>• living in dorms<br>• procrastination
Hussain, 2018 [62]	No	None	Female: 56.7%<br>Male: 43.3%	IA Prevalence: 41.7%.	No Acknowledged in Introduction.<br><br>Academic figures not mentioned in Discussion.	Internet mainly used for:<br>• entertainment: 38.3%[a]<br>• social: 33.3%<br>• *research/academics: 15.8%*<br>• other: 12.5%

Lead Author, Publication Date, Citation	Validity of IA questioned?	Measured against (e.g. Depression	Age & Gender	Addiction rates (Prevalence)	Mentioned value of Internet for Academic work	Results
						IA correlated with:<br>• year of study (2nd > 3rd)<br>• Type B personality > Type A
Ja'ffar, 2019 [63]	No	None	Female: 59.32%[a]<br>Male: 40.68%[a]	IA:<br>None: 10.6%<br>Mild: 40.3%<br>Moderate: 45.6%<br>Severe: 3.4%	Acknowledged in Introduction.<br><br>Mentioned only<br><br>No significant association between IA and educational use, but the fact that 56% of the students use it for educational purposes ignored in the interpretation.	Internet used for:<br>• social network: 89.7<br>• education: 56.3%<br>• gaming: 30.4<br>• e-mail: 20.2<br><br>IA Correlated with:<br>• hours spent online per day<br><br>No correlations with: gender, parents' marital status, academic grade, residence and parents' level of education, years of internet use, gaming.
Jain, 2018 [64]	No	None	Female: 49.4%<br>Male: 50.6%	IA:<br>Normal: 6.7%<br>Mild: 38.7%<br>Moderate: 49.3%<br>Severe: 5.3%	Acknowledged in Introduction.<br><br>High use for Academic work ignored in the interpretation.	Internet used for:<br>• chatting: 100%<br>• YouTube: 100%<br>• online friendship/ relationship: 94%<br>• *academic work: 69.3%*

Lead Author, Publication Date, Citation	Validity of IA questioned?	Measured against (e.g. Depression	Age & Gender	Addiction rates (Prevalence)	Mentioned value of Internet for Academic work	Results
						• movies: 62.7%<br>• shopping: 58%<br>• games: 41.3%<br>• pornography: 14.7%<br><br>IA correlated with:<br>• staying in hostel and living single<br>• usage of smart phone<br>• preferring online shopping<br>• preferring online studying.<br><br>No correlation with gender.
Javaeed, 2019 [65]	No, but mentions that it is not in DSM.	DASS21 for depressi oNone	Female: 64.3%[a]<br>Male: 35.7%	IA:<br>None: 3.3.%<br>Mild: 44.3%<br>Moderate: 51.0%<br>Severe: 1.4%	Acknowledged in the Introduction.<br><br>No further mention of Internet for work-related activities.	IA Correlated with:<br>• depression<br>• stress
Javaeed, 2020) [66]	No	None	Female: 59.2%<br>Male: 40.8%[a]	IA:<br>None: 0.9%<br>Mild: 12.0%<br>Moderate: 58.9%<br>Severe: 28.2%	Yes, in Introduction. No further mention of value to work.	IA correlated with:<br>• year of study (third year students scoring the highest)

**Lead Author, Publication Date, Citation**	**Validity of IA questioned?**	**Measured against (e.g. Depression**	**Age & Gender**	**Addiction rates (Prevalence)**	**Mentioned value of Internet for Academic work**	**Results**
						IA negatively correlated with:<br>• academic performance<br><br>IA did not correlate with age or gender.
Kannan, 2019 [67]	No	Auditory reaction time-ART, visual reaction time VRT Heart Rate Variability (HRV)	Female: 36.8%<br>Male: 63.2%	IA:<br>Not: 82.6%[a]<br>Addict: 17.4%	No mention of it in the paper.	Auditory reaction time was significantly prolonged in the internet addicts compared to the non-addicts.<br><br>IA correlated with:<br>• gender (M>F)
Kapadia, 2015 [68]	No	None	Mean age: 21.75±2.96<br>Female: 37%<br>Male: 63%	IA:<br>Normal: 17.5%<br>Mild: 62.5%<br>Moderate: 20%<br>Severe: 0%	No, Not mentioned in Introduction.<br><br>Results of high use for study repeated in the Discussion, but no	Internet used for:<br>• entertainment: 83.5%<br>• *study: 80.5%*<br>• chatting: 80.5%<br>• email: 61.5%<br>• online shopping: 61%<br>• *research: 49%*

Lead Author, Publication Date, Citation	Validity of IA questioned?	Measured against (e.g. Depression	Age & Gender	Addiction rates (Prevalence)	Mentioned value of Internet for Academic work	Results
					significance of that figure made.	
Khan, 2016) [69]<br>Khan, 2017) [70]	No	Academic performance	Female: 45.7%<br>Male: 54.3%	IA:<br>Normal: 83.2%<br>Moderate: 16.1%<br>Severe: 0.6%	Acknowledged in Introduction of both papers.<br><br>No mention of it in the Discussion.	IA correlated negatively with:<br>• academic performance.<br>• physical activity
Komleh, 2015 [71]	No	academic achievement (GPA)	Female: 58%<br>Male: 42%	IA<br>15.2% had IA (taken as > 44 on the scale).	No mention in Introduction.<br><br>No measure of activities, although the discussion does note the impact on critical thinking and lower failure rate, but still does not reinterpret the "addiction" rates.	IA correlated with :<br>• time spent on the Internet<br><br>IA inversely correlated with<br>• failure of credits (higher IA, fewer failures).<br><br>No correlation with gender, GPA
Kootesh, 2016 [10]	No, and indicates that it is in DSM-5	Pittsburgh Sleep Quality Inventory (PSQI) Mental Health	Female: 52%<br>Male: 48%	IA Mean: (37.17±13.40)	No mention in the Introduction or Discussion.	IA negative correlated with:<br>• sleep quality<br>• mental health

**Lead Author, Publication Date, Citation**	**Validity of IA questioned?**	**Measured against (e.g. Depression**	**Age & Gender**	**Addiction rates (Prevalence)**	**Mentioned value of Internet for Academic work**	**Results**
		Checklist (MHC)				
Kumar, 2017 [72]	No	None	Mean age: 20.67±1.08<br>Female/Male: 50/50	IA<br>No addiction: 60%<br>Mild: 30%<br>Moderate: 10%	Acknowledged in Introduction.<br><br>No mention of work in the Discussion, so the 61% using it for study purposes ignored.	Internet used for:<br>• entertainment: 62.7%<br>• *study: 61.3%*<br>• keeping in touch with friends and family: 61.3%<br><br>IA correlated with:<br>• gender (M>F)<br>• hours of internet usage per day<br>• expenditure on Internet
Kundu, 2017 [73]	No	Becks depression scale	Mean age: 20.15±1.89<br>Female: 75.38%<br>Male: 24.62%	IA:<br>None: 17.6%<br>Mild: 59.23%<br>Severe: 23.08%	Introduction: Mentions use of internet by health professionals for work, but not about students for studying.<br><br>Makes no reference to the high academic percentage in the results.	Internet used for:<br>• social media: 73.08%<br>• downloading media files: 65.38%<br>• academic purposes: 63.08%<br>• shopping: 51.54%<br><br>IA correlated with:<br>• gender (M>F)

Lead Author, Publication Date, Citation	Validity of IA questioned?	Measured against (e.g. Depression	Age & Gender	Addiction rates (Prevalence)	Mentioned value of Internet for Academic work	Results
						IA negatively correlated with:<br>• depression
Latt, 2017 [74]	No	Islamic principles	Female: 64.1%<br>Male: 35.9%	IA:<br>Normal: 5.8%, 48.5% mild, 44.7 moderate, 1% severe	Acknowledged in the Introduction.<br><br>Some of the academic-related benefits of the Internet are explored in the Discussion, but does not impact on interpretation of the addiction rates. However, it gives a more balanced view than most of the other papers.	
Liu, 2009 [75]	No	Academic performance	Female: 48.9%[a]<br>Male: 51.1%[a]	Prevalence of IAD:<br>Female: 11.6%<br>Male: 20.6%	Acknowledged in Introduction<br><br>The high proportion of work-related activity is noted in the Discussion, and appears to moderate their authors fears of addiction.	"Frequent" use of Internet:<br>• email: 51.4%<br>• searching for information: 34.4%<br>• chatting: 29.3%<br>• news: 25.0%<br>• entertainment news: 24.1%<br>• music: 23.0%<br>• films: 20.7%

Lead Author, Publication Date, Citation	Validity of IA questioned?	Measured against (e.g. Depression	Age & Gender	Addiction rates (Prevalence)	Mentioned value of Internet for Academic work	Results
						IA correlated with:<br>• gender (M>F)<br><br>IA negatively correlated with:<br>• academic performance.
Loredo e Silva, 2018 [76]	No	Biggs' Study Process Questionnaire (R-SPQ-2F):	Mean age: 22.11±3.11<br>Female: 55.4%<br>Male: 44.6%[a]	IA Mean score: 46.27 (SD:12.12);<br>Non-problematic: 31.8%<br>Problematic: 68.2% (Frequent= 64.6%; significant= 3.6%)	No. Mentions educational value of smartphones, but not Internet (it might be implied)	Had medical apps: 67.8% (of the clerkship students, this rose to 93.4%)<br>Use phone apps for learning: some 30-50% of the students.<br><br>IA correlated with:<br>• surface learning approaches<br>• lower scores in deep learning approaches
Madhusudan, 2018 [77]	No, but mentions that it is not in	None	Mean age: 21.66±1.59<br>Female: 65.6%<br>Male: 34.4%	IA:<br>None: 5.5%<br>Mild: 60.8%<br>Moderate: 31.3%<br>Severe: 2.5%	Acknowledged in the Introduction.<br><br>No mention of the 20% used "to gain knowledge"	Internet used for:<br>• social networking: 55.93%<br>• communication: 21.81%<br>• *gain knowledge: 20.58%*<br>• others: 1.63%

Lead Author, Publication Date, Citation	Validity of IA questioned?	Measured against (e.g. Depression	Age & Gender	Addiction rates (Prevalence)	Mentioned value of Internet for Academic work	Results
	the DSM.				and any modification of addiction. Also, the Communication is ill-defined, and may be work-related or not.	IA correlated with:<br>• gender (M>F)<br>• phase of degree (Final>others)<br>• place of origin (urban>rural)<br><br>IA negatively correlated with:<br>• previous marks obtained
Mallya, 2019 [78]	No	Depression, anxiety, and stress scale 21; Heart rate and BP components such as systolic and	Age: 19.56[a]<br>Female: 61%<br>Male: 39%	IA rate: 44%<br>(Female: 52%<br>Male: 31%)	Acknowledged in the Introduction.<br><br>No mention of work in the Discussion.	Internet used mainly for:<br>• social media: 85%<br>• gaming: 10%<br>• academic: 4%<br>• shopping: 2%<br><br>IA correlated with:<br>• stress<br>• depression

Lead Author, Publication Date, Citation	Validity of IA questioned?	Measured against (e.g. Depression	Age & Gender	Addiction rates (Prevalence)	Mentioned value of Internet for Academic work	Results
		diastolic BP				
Malviya, 2014 [79]	No	None	Age range: 21-25.<br>Female: 32.2%<br>Male: 67.8%	IA:<br>None: 7.4%<br>Average: 64.5%<br>Moderate: 18.6%<br>Severe: 9.5%	No mention of work in the Introduction, or anywhere else in the paper.	No correlation with gender.
Mohammadbeigi, 2016 [80]	No	Self-Rated Health	Mean age: 21.7±2.9<br>Female: 84.3%<br>Male: 15.7%[a]	28.7% were addicted; mean score: 35.22±12.1.	Yes, in the Introduction. The high percentage usage for work-related activities is not raised in the Discussion.	64.2% use it for "scientific search" and 37.0% use it for "Academic research".<br><br>IA correlated with:<br>• Gender (F>M)<br>• Entertainment<br>• Chat rooms<br>• Have email<br>• Use of Internet<br><br>IA negatively correlated with:<br>• Self-reported Health<br>• academic performance
Mostafa, 2019 [81]	No	None	Mean age: 21.78±1.48<br>Female: 74.0%	IA:<br>Mild: 63.69%<br>Moderate: 35.43%<br>Severe: 1.18%	Discussed in some detail in the Introduction.	Internet used for:<br>• social networking: 86.61%<br>• *education: 66.53%*

Lead Author, Publication Date, Citation	Validity of IA questioned?	Measured against (e.g. Depression	Age & Gender	Addiction rates (Prevalence)	Mentioned value of Internet for Academic work	Results
			Male: 26.0%		Mention of it in the Discussion but no interpretation impact on the Results	• entertainment: 61.81%<br>• e-mail communication: 21.25%<br>• *research: 9.05%*<br>• net meeting: 5.51%<br>• others: 1.57%
Mukherjee, 2020 [82]	No	Insomnia Severity Index; Rosenberg Self Esteem Scale (RSES)	Mean age: 20.0[a]<br><br>Female: 48.7%<br>Male: 51.3%	Average IA score: 51.76 ± 16.96<br><br>IA:<br>"Average" (20-39): 30.0%<br>"over-users" (40-69): 50.7%<br>"internet addicts" (70-100): 19.3%	Acknowledged in the Introduction.<br><br>No mention of work in the paper.	IA correlated with:<br>• gender (M>F)<br><br>IA correlated negatively with:<br>• self-esteem
Nagori, 2016 [83]	No	Beck's Anxiety Inventory (BAI); World health organization quality of life	Female: 53.33%<br>Male: 46.67%	IA:<br>Less than average: 34.3%<br>Average: 56.4%<br>Moderate: 8.4%<br>Addict: 0.9%	Acknowledged in Introduction.<br><br>No mention of the high use of Internet for academic purposes in the Discussion.	Internet mainly used for:<br>• social networking: 81.5%<br>• *education: 81.3%*<br>• entertainment: 72.2%<br><br>IA correlated with:<br>• gender (M>F)

**Lead Author, Publication Date, Citation**	**Validity of IA questioned?**	**Measured against (e.g. Depression**	**Age & Gender**	**Addiction rates (Prevalence)**	**Mentioned value of Internet for Academic work**	**Results**
		assessment scale (WHOQOL-BREF).				
Nath, 2016 [84]	No, although notes that Goldberg introduced it as satire, and that it is not in DSM 5.	None	Mean age: 22.51±2.91<br>Female: 39.4%<br>Male: 60.6%	IA:<br>Less than average: 9.04%<br>Average: 44.15%<br>Possible addict: 46.28<br>Addict: 0.53	Acknowledged in Introduction.<br><br>No mention of academic work in Discussion.	Internet used for:<br>• social networking: 81.5%<br>• *education: 81.3%*<br>• entertainment: 72.2%<br>• social networking: 67.0%<br>• academic content: 13.3%<br>• downloadable media: 9.6%<br>• social media: 8.5%<br>• pornography: 1.6%.<br><br>IA correlated with:<br>• gender (M>F)<br>• preferred to always stay online<br>• exposed to the Internet for 6 years or more<br>• forming online relationships

Lead Author, Publication Date, Citation	Validity of IA questioned?	Measured against (e.g. Depression	Age & Gender	Addiction rates (Prevalence)	Mentioned value of Internet for Academic work	Results
						• poor performance at college<br>• feelings of anxiety and depression
Öztürk Kaygusuz, 2019 [85]	No, although says it is not in the DSM.	None	Mean age: 20.47±1.88<br>Female: 52.6%<br>Male: 47.4%[a]	IAT Mean Score: 29.92±16.33<br><br>Not Addicts: 77.5%<br>Probable Addicts: 19.4%<br>Addicts: 3%	Not in introduction.<br><br>Results that show correlation to work noted in the Discussion, yet has no impact on the interpretation of the results.	IA correlated with:<br>• time on Internet<br>• *internet use for information search*<br>• *course-training-research*<br>• video conversation<br>• suffering from sleeplessness
Patel, 2018 [86]	No	None	Mean age: 19.4.<br>Female: 41.72%<br>Male: 58.28%	Possible Addicts: 74.82%<br>Severe: 16.55%<br>Mild: 8.63%	In the Introduction, mentions that it is useful for "knowledge sharing"<br><br>Academic use results ignored in the Discussion	Academic purposes bundled with gaming, social media, so it not possible to extract.
Patil, 2017 [87]	No	None	Mean age: 20<br>Female: 43.65%<br>Male: 56.35%	IA:<br>Average: 61.47%<br>Possible: 34.83%<br>Addicts: 3.68%	Acknowledged in Introduction.<br><br>No mention in paper of use of Internet for academic work	IA correlated with:<br>• gender (M>F)

Lead Author, Publication Date, Citation	Validity of IA questioned?	Measured against (e.g. Depression	Age & Gender	Addiction rates (Prevalence)	Mentioned value of Internet for Academic work	Results
Pramanik, 2012 [88]	No	None	Female: 50%<br>Male: 50%	IA:<br>Mild: 40%<br>Moderate: 41.53%<br>Severe: 3.05%<br>None: 15.42%[a]	Acknowledged in Introduction.<br><br>No measure of educational usage; no discussion of educational usage in the paper.	
Qadir, 2018 [89]	No	None	Mean age: 22.02[a]<br>Females: 50%<br>Males: 50%	IA:<br>Normal: 53%<br>Problematic: 45%<br>Addict: 2%	No mention in the Introduction.<br><br>The fact that 20% used it mainly for study does not influence the interpretation.	Internet mainly used for:<br>• entertainment: 53%<br>• study: 20%<br>• information: 15%<br>• communication: 9%<br>• gaming: 2%<br>• shopping: 1%
Radeef, 2018 [90]	No, although noted that it is not included in DSM-5. Compared to Internet	Depression Anxiety, Stress Scale (DASS-21)	Mean age: 22.87 years<br>Female: 30.6%<br>Male: 69.4%[a]	22.8% were IA	Acknowledged in the Introduction.<br><br>No further mention of educational work in their paper.	IA correlated with:<br>• depression<br>• anxiety symptoms<br><br>IA negatively correlated with:<br>• social interaction (inverse)

**Lead Author, Publication Date, Citation**	**Validity of IA questioned?**	**Measured against (e.g. Depression**	**Age & Gender**	**Addiction rates (Prevalence)**	**Mentioned value of Internet for Academic work**	**Results**
	gaming disorder					
Rebello, 2017 [91]	No	None	Female: 42.6%[a]<br>Male: 57.4%[a]	CUI prevalence: 11.21%	Acknowledged in Introduction.<br><br>Academic results referred to in Discussion, but no implications on the CUI of this discussed.	Internet used for:<br>• *Studies: 96.1%*<br>• Get connected with people: 90.6%<br><br>Felt:<br>• Technology should be utilized: 96.1%<br>• Usage can be restricted: 81.3%<br>• Internet usage as distraction: 58.9%<br>• Hinder academics: 47.2%<br><br>CUI correlated with:<br>• mobile usage duration<br>• Internet usage duration<br>• maximum usage time<br>• gender (M>F)
Rustam, 2017 [92]	No, although mentions that it is	None	Age range: 18-25.<br>Female: 53%<br>Males: 47%	IA:<br>Normal: 27%<br>Mild: 45.5%<br>Moderate: 27%	No mention in the Introduction.	Only IA results reported.

**Lead Author, Publication Date, Citation**	**Validity of IA question ed?**	**Measur ed against (e.g. Depress ion**	**Age & Gender**	**Addiction rates (Prevalence)**	**Mentioned value of Internet for Academic work**	**Results**
	not in DSM.			Severe: 0.5%	No mention of work or academics in the paper.	
Sahraian, 2016 [93]	No	Personal ity (NEO-FFI)	Mean age: 21.48±2.59<br>Female: 61%<br>Male: 39%	IA<br>Normal: 45.3%<br>Mild: 51.4%<br>Moderate: 2.9%<br>Severe: 0.4%	Yes, mention academic work on Internet.<br><br>Academic work, including the high percentage in these results, ignored in the Discussion	Internet used for:<br>• *scientific research: 64.0%*<br>• social web: 63.3%<br>• e-mail: 59%<br>• non -scientific research: 57%<br>• music: 53.2%<br>• film: 41%<br>• software: 41%<br>• chat: 30.2%<br>• news: 39.2%<br>• gaming: 17.6%<br><br>IA correlated with:<br>• neuroticism<br>• gender (M>F)<br>• live with family > Residence<br>• single > married<br><br>IA negatively correlated with:<br>• extraversion

Lead Author, Publication Date, Citation	Validity of IA questioned?	Measured against (e.g. Depression	Age & Gender	Addiction rates (Prevalence)	Mentioned value of Internet for Academic work	Results
						• agreeableness<br>• conscientiousness
Saied, 2016) [94]	No	None	Mean age: 22.08±1.15<br><br>Female: 56.56%[a]<br>Male: 43.44%[a]	IA:<br>Average: 64.1%<br>Potentially addicted: 33.2%<br>Addicted: 2.7%	No. The high usage figure of the Internet for academic purposes ignored in the Discussion.	Internet used for[a]:<br>• Facebook: 92.0%<br>• *information & research: 80.0%*<br>• other social media: 77.0%<br>• entertainment: 76.9%<br>• *studying: 72.9%*<br><br>Of those who used it for Facebook, 79.1% used it for educational purposes.<br><br>IA correlated negatively with:<br>• academic grades<br><br>Adverse effects: eye irritation, followed by headache, then back pain
Saini, 2016 [95]	No	Personality Traits Goldberg's Big-Five	Female: 36.6%<br>Male: 63.4%	Mean IA score: 33.94 (SD 13.592)<br>No other IA figures given.	No mentioned in the Introduction.<br><br>No mention of work or academics in paper.	IA correlated with:<br>• higher neuroticism<br>• less extroversion

**Lead Author, Publication Date, Citation**	**Validity of IA questioned?**	**Measured against (e.g. Depression**	**Age & Gender**	**Addiction rates (Prevalence)**	**Mentioned value of Internet for Academic work**	**Results**
		factor test				
Salehi, 2014 [96]	No	None	Mean age: 21.79±2.42<br>Female: 61.1%<br>Male: 38.9%	Not addicted: 92.7%<br>At risk: 2.1%<br>Addicted 5.2%	No mention in the Introduction.<br><br>Although scientific research result referred to in the Discussion this did not impact on the interpretation of data.	Internet used for<br>• email: 54%<br>• *scientific research: 39.9%*<br>• communicating with friends and family: 35%<br>• download films and music: 33.7%<br>• news: 25.1%<br>• chatting: 6.3%<br>• shopping: 5.7%<br>• writing blogs: 5.7%<br>• playing games: 2.9%<br><br>IA correlated with:<br>• Gender (M>F)<br>• Education stage<br>• Time spent of Internet<br>• Games<br>• Communicating with friends and family
Salek Ebrahimi, 2019 [97]	No	General Self-efficacy,	Mean age: 19.73±1.11	IA<br>Normal (<50): 82.9%	No mention of educational work in the paper.	IA Correlations unclear and contradictory.

Lead Author, Publication Date, Citation	Validity of IA questioned?	Measured against (e.g. Depression	Age & Gender	Addiction rates (Prevalence)	Mentioned value of Internet for Academic work	Results
		Difficulty in Emotion Regulation, and Resilience	Female: 47.9%<br>Male: 52.1%	At risk (50-79): 15.6%<br>Dependent (80-100): 2.1%		
Samaha, 2019 [98]<br>Samaha, 2018 [99]	No, although acknowledges that there is currently no "gold standard" tool for measurement.	Medical Student Stressor Questionnaire	Mean age: 21.92±2.16<br>Female: 63.4%<br>Male: 36.6%	IA:<br>Normal: 25.2 %<br>Mild: 48.7 %<br>Moderate: 24.8%<br>Severe: 1.3%	No mention in the Introduction.<br>No mention in the paper. One paper is data; the focus was on the internal consistency of the IAT.	IA negatively correlated with:<br>• income<br><br>No correlation on gender
Şenol, 2019 [100]	No	Sagittal alignment (Cobb)	Age range: 18-22<br>Female: 67.3%<br>Male: 32.7%	IA:<br>Not Addicted: 55.4%<br>"Mean addicted": 39.3%<br>Addicted: 5.4%	No, although it does mention quick access to "scientific resources" in general.	Internet used for<br>• "research, lessons, news, music, video and social nets": "about 85%"<br>• games: 7.1%

Lead Author, Publication Date, Citation	Validity of IA questioned?	Measured against (e.g. Depression	Age & Gender	Addiction rates (Prevalence)	Mentioned value of Internet for Academic work	Results
					Does take it into account a little, but still equates usage with addiction.	IA negatively correlated with:<br>• lumbar Cobb angle
Shadzi, 2020 [101]	No	Depression Anxiety Stress Scale (DASS-21); Pittsburgh Sleep Quality Index (PSQI)	Mean age: 22.4±2.18<br><br>Female: 50.3%<br>Male: 49/7%[a]	38.6% had problematic internet use	Not mentioned in Introduction or anywhere else in paper.	Problematic internet use predicted poor sleep quality through indirect pathways by the mediation of mental health problems.
Shi, 2019 [102]	No (in the Discussion, the authors do note that no IA is the gold standard.	Big Five Inventory (BFI); ADHD Self-Report Scale-V1.1;	Mean age: 19.74±1.48<br>Female: 58.9%<br>Male: 41.1%	IA:<br>None: 55.3%[a]<br>Mild: 35.5%<br>Moderate: 8.6%<br>Severe: 0.6%	No mention in paper of value to students.<br><br>Generally high usage levels seen in opposition to work: "They are self-disciplined, diligent and goal striving, so that they can have better control of Internet use, and refrain	IA correlated with:<br>• age group<br>• academic year<br>• hometowns<br>• neuroticism<br><br>IA negatively correlated with:<br>• extraversion<br>• agreeableness<br>• conscientiousness

Lead Author, Publication Date, Citation	Validity of IA questioned?	Measured against (e.g. Depression	Age & Gender	Addiction rates (Prevalence)	Mentioned value of Internet for Academic work	Results
					from becoming addicted to it [i.e. the Internet].”	• openness
Shinde, 2018 [103]	No	DASS 21 (Depression)	Mean age cannot be calculated because of conflicting results.<br>Female: 43.33%<br>Male: 56.66%	IA:<br><30: 31.11%<br>30-49: 48.89%<br>50-79: 20%<br>80-100: 0%	Acknowledged in the Introduction.<br><br>No examination of academic or other work; no mention anywhere in the paper.	IA correlated with:<br>• depression
Shoghli, 2018) [6]	No	General health questionnaire	Mean age: 25.29±1.23<br>Female: 51.1%	IA<br>Regular users: 63.5%<br>High risk: 36.5%	Nothing about using the Internet for academic work.	IA correlated with:<br>• marital status (S>M)<br>• maternal occupation<br>• maternal education

Lead Author, Publication Date, Citation	Validity of IA questioned?	Measured against (e.g. Depression	Age & Gender	Addiction rates (Prevalence)	Mentioned value of Internet for Academic work	Results
		(GHQ-28)	Male: 48.9%	Addict: 0%		IA negatively correlated with:<br>• general health
Simcharoen, 2018 [104]	No	Loneliness, interpersonal problems UCLA loneliness scale; Inventory of Interpersonal Problems-32	Mean age: 20.88±1.8<br>Female: 56.8%<br>Male: 43.2%[a]	IA:<br>None: 63.3%<br>Mild: 30.9%<br>Moderate: 5.2%<br>Severe: 0.6%	Acknowledged in the Introduction.<br><br>No mention of academic work elsewhere in the paper.	Internet used for<br>• social networking: 4.52%<br>• chatting 4.46%<br>• YouTube 4.28%<br>• searching for information (e.g. Google) 4.27%<br>• movies/music 3.42%<br>• downloading (movies/music etc.) 3.36%<br>• *reading e-learning 3.08%*<br>• emailing 2.65%<br>• shopping/auction 2.09%<br>• forum participation (Web board): 1.96%<br>• gaming 1.92%<br>• banking/business: 1.56%<br>• blogging/diary: 1.22%<br>• gambling 1.04%
Singh, 2018 [105]	No (althoug h	Epworth Sleepine	Mean age: 21.9±4.6 years	IA<br>Normal: 34.23%<br>Mild: 57.72%	No mention in the Introduction.	IA correlated with:<br>• excessive daytime sleepiness

Lead Author, Publication Date, Citation	Validity of IA question ed?	Measur ed against (e.g. Depress ion	Age & Gender	Addiction rates (Prevalence)	Mentioned value of Internet for Academic work	Results
	acknowl edges that it is not in DSM)	ss Scale (ESS)	Females: 71.82%<br>Males: 28.18%	Moderate: 7.38%<br>Severe: 0.67%	No mention of academic work in the paper.	
Singh, 2018 [106]	No, but does acknowl edge that "Measur ement of level of problem atic internet use has been a challeng e"	Problem atic Internet behavio urs.	Mean age: 20.6±0.88<br>Female: 21.3%[a]<br>Males: 78.7%	IA:<br>Average: 80.3%<br>Moderate: 19.7%<br>Severe: 0%	No mention of work. But they state this:<br>Find higher Internet usage in the classroom among those with higher IA scores, but do not consider the possibility that this usage may be work-related.	IA correlated with:<br>• use of email<br>• social networking<br>• blogging<br>• forums<br>• online leisure activities<br>• surfing without purpose<br>• online shopping<br>• downloading
Siraj, 2015 [107]	No	cGPA	Female: 73%<br>Males: 27%	Internet user > 6 hours were observed to have higher CGPA	Discussed in some detail in Introduction.<br><br>Quite a bit of discussion on the topic. Does not explicitly modify the interpretation of the	Internet used for:<br>• *course-related: 84.1 %*<br>• entertainment: 76.1%<br>• *assignment: 71.6%*<br>• non-course related: 43.2%<br>• chat: 38.1%<br>• email: 35.8%

Lead Author, Publication Date, Citation	Validity of IA questioned?	Measured against (e.g. Depression	Age & Gender	Addiction rates (Prevalence)	Mentioned value of Internet for Academic work	Results
					addiction figures, but does somewhat put it into perspective.	• music: 33.5%<br>• play game: 11.4%<br>• download game: 4.5%<br>• create web page: 1.7%
Srijampana, 2014 [108]	No, although does acknowledge that there is a debate about how best to classify it.	None	Mean age: 19.9<br>Female: 57.2%<br>Male: 42.8%	Less than average: 23.2%<br>Average users: 64.4%<br>Possible: 11.8%,<br>Addicts: 0.4%	Acknowledged in Introduction.<br><br>Incomplete/contradictory data on academic usage.	Internet used for:<br>• social networking: 59.7%<br>• downloading media files: 18.9%<br>• online gaming: 12.3%<br>• *academic purposes: 9.0%*<br><br>Correlation only of social media sites with gender (M>F).
Subhaprada, 2017 [109]	No	Academic performance (self-reported, no details)	Females: 37.8%<br>Males: 62.2%	IA:<br>None: 23.16%<br>Mild: 52.63%<br>Moderate: 24.21%<br>Severe: 0%	Acknowledged in the Introduction, no mention in discussion	IA correlated with:<br>• gender (M>F)<br><br>IA negatively correlated with:<br>• academic performance.
Suresh, 2018 [110]	Yes, on definitio	Lyubomirsky		IAT:<br>None: 12.6%	Acknowledged in the Introduction.	Higher levels of internet addiction showed lower

Lead Author, Publication Date, Citation	Validity of IA questioned?	Measured against (e.g. Depression	Age & Gender	Addiction rates (Prevalence)	Mentioned value of Internet for Academic work	Results
	n and whether it should be included in DSM.	and Lepper's Subjective Happiness Scale		Mild: 58.2%<br>Moderate: 28%<br>Severe: 0.7%[a]	No mention in Discussion, although the Conclusion does point out that the Internet is important for academic work. This, however, does not influence the interpretation of the results.	levels of subjective happiness
Sushma, 2018 [111]	No	None	Mean age: 20.6±1.97<br><br>Female: 37.29%<br>Male: 62.17%	IA:<br>None: 21.2%<br>Mild: 58.2%<br>Moderate: 19.5%<br>Severe: 0.8%	Not mentioned in Introduction<br><br>No mention in the Discussion.	Internet used for:<br>• social networking: 25%<br>• media: 24.20%<br>• *academics: 15.3%*<br>• games: 10.2%<br>• others: 25.4%<br><br>IA correlated with:<br>• gender (M>F)<br>• time spent on using internet per day
Taha, 2019 [11]	No Incorrectly states that it is included	None	Females: 42.1%[a]<br>Males: 57.9%	IA:<br>Lower than average: 2.9%<br>Average: 26.8%	Not mentioned in Introduction.<br><br>In limitations, does acknowledge that "some	IA correlated with:<br>• gender (F>M)<br>• year of study: 2nd year > 1st and 3rd year

Lead Author, Publication Date, Citation	Validity of IA questioned?	Measured against (e.g. Depression	Age & Gender	Addiction rates (Prevalence)	Mentioned value of Internet for Academic work	Results
	in the DSM.			Possible addict: 57.9%<br>Addict: 12.4%	of the students may have been using the Internet for work- or research-related purposes", but this does not ever affect the interpretation of their results as "addiction"	• neck pain<br>• sleeplessness<br><br>Problems also reported: headaches, backache, weight gain, neck pain and other psychological problems
Tan, 2019 [7]	No, acknowledges that it is not in the DSM, but suggests that it should be.	UCLA Loneliness Scale; Academic Expectations Stress Inventory; Multidimensional Scale of Perceived Social Support	Mean age: 21.01±1.09<br>Female: 70%<br>Male: 30%	IA: 31.9%	Not mentioned in the paper.	IA negatively correlated with:<br>• social support from family<br>• social support from friends<br>• social support from significant other

**Lead Author, Publication Date, Citation**	**Validity of IA questioned?**	**Measured against (e.g. Depression**	**Age & Gender**	**Addiction rates (Prevalence)**	**Mentioned value of Internet for Academic work**	**Results**
Tsimtsiou, 2014 [112]	No	None	Mean age: 21.1±3.5	Mean IAT score: 31.2 (SD = 16.4)<br>Normal: 52.9 %<br>Mild: 31.4 %<br>Moderate: 15.7 %	No discussion of Internet for work purposes anywhere in the paper.	Internet used for:<br>• gambling: 39.1%<br>• gaming: 35.1%<br>• adult entertainment: 35.1%<br><br>IA correlated with:<br>• gambling<br>• pornographic sites<br>• games
Tsimtsiou, 2015 [113]	No	None	Mean age: 21.34[a]	IA:<br>IA Mean score: 25.4 (SD=13.9)<br>Normal: 69.9%<br>Mild: 24.5%<br>Moderate: 5.4%<br>Severe: 0.2%	Not mentioned anywhere in the paper; even the fact that 76% of the students use the Internet for their education is ignored in the Discussion.	Internet used for:[a]<br>• Facebook: 80.15%<br>• e-mail: 77.90%<br>• *education: 75.84%*<br><br>IA correlated with:<br>• visits in Internet cafes<br>• *at school*<br>• via mobile application<br>• Facebook,<br>• Twitter<br>• online games<br>• mean duration of Internet use

**Lead Author, Publication Date, Citation**	**Validity of IA questioned?**	**Measured against (e.g. Depression**	**Age & Gender**	**Addiction rates (Prevalence)**	**Mentioned value of Internet for Academic work**	**Results**
Upadhayay, 2017 [114]	No	None	Females: 50%<br>Males: 50%	IA:<br>None: 79%<br>"Slightly Addicted": 21%	Not mentioned in Introduction.<br><br>Use of Internet for education acknowledged in the Discussion, although makes the point that none of those who said it was a priority falls into the Addicted group.	Highest priority to using the Internet:<br>• movies/songs: 18%<br>• *educational and learning activities: 17%*<br>• communicating with friends and families: 14%
V, 2017 [115]	No	Depression, anxiety and stress (DASS 42).	No age or gender data given.	IA[a]:<br>None: 29.7%<br>Mild: 49.1%<br>Moderate: 19.1%<br>Severe: 2.0%	No mention made of work anywhere in the paper.	IA correlated with:<br>• anxiety levels
Vidyachathoth, 2013 [116]	Does acknowledge that there was some debate prior to DSM-5	Affect (PANAS)	Mean age: 18.49±0.71<br><br>Female: 62.2%<br>Males: 37.8%		No mention in the Introduction.<br><br>Only leisure work studied, so no mention of work.	IA negatively correlated with:<br>• Affects score.

Lead Author, Publication Date, Citation	Validity of IA questioned?	Measured against (e.g. Depression	Age & Gender	Addiction rates (Prevalence)	Mentioned value of Internet for Academic work	Results
Wang, 2020 [117]	No	Pittsburgh Sleep Quality Index (PSQI); Self-Harm Questionnaire	Mean age: 18.8[a]<br>Female: 58.48%<br>Male: 41.52%	28.2% reported having IA (IA score > 40).	Not mentioned in Introduction.<br><br>Use of the Internet for work ignored.	IA correlated with:<br>• gender (F>M)<br>• upper grade students<br>• poor sleep quality<br>• possibility of self-injury
Yerpude, 2019 [118]	No	Mental health inventory	Mean age: 19.32±1.64<br><br>Female: 58.33%<br>Male: 41.67%	Prevalence: 23.48%	Acknowledged in the Introduction.<br><br>No mention of work-related activities in the paper.	Internet used for:<br>• social networking: 35.61%<br>• *educational: 25.76%*<br>• recreational: 21.21%<br>• games: 17.42%<br><br>IA correlated with:<br>• gender (M>F)<br>• computer ownership<br>• login status<br>• mode of internet access<br>• anxiety<br>• depression

Lead Author, Publication Date, Citation	Validity of IA questioned?	Measured against (e.g. Depression	Age & Gender	Addiction rates (Prevalence)	Mentioned value of Internet for Academic work	Results
						• loss of emotional/behavioral control<br>• emotional ties<br>• psychological distress
Yücens, 2018 [119]	Yes; indicates that it is not in the DSM, and other indications that definition is difficult. Also, that IA may be some form of self-medication	Liebowitz Social Anxiety Scale (LSAS); Barratt Impulsivity Scale-11 (BIS-11) Rosenberg Self-Esteem Scale (RSES); Beck Depression Inventory (BDI);	Female: 57.4%[a]<br>Male: 42.6%[a]	IA<br>None (<50): 73%<br>Moderate: 23.7%<br>Severe: 3.3%	Acknowledged in Introduction<br><br>Not mentioned anywhere in the paper.<br><br>In spite of the caveats in the Introduction, this does not appear to affect the interpretation of addiction rates.	IA correlated with:<br>• anxiety (including social anxiety)<br>• depression<br><br>IA negatively correlated with:<br>• self-esteem

Lead Author, Publication Date, Citation	Validity of IA questioned?	Measured against (e.g. Depression	Age & Gender	Addiction rates (Prevalence)	Mentioned value of Internet for Academic work	Results
	against other problems. It may resemble ICD more than an addiction	Beck Anxiety Inventory (BAI).				

[a] Not stated in the paper, but calculated based upon the other percentages and raw data.
[b] The authors did not use the standard classification; instead, they used None (<40), Possible (40-69), Addicted:>70
