# Supplementary material for: How Have Researchers Acknowledged and Controlled for Academic Work Activity When Measuring Medical Students’ Internet Addiction? A Systematic Literature Review
Source: Int J Environ Res Public Health. 2021 Jul 20;18(14):7681. doi: 10.3390/ijerph18147681 (PMC8306379; doi:10.3390/ijerph18147681)
Supplement: Supplementary file 1 [file ijerph-18-07681-s001.zip › TableS2.pdf]

Table S2: Detailed Results

**Lead Author, Publication Date, Citation**	**Validity of IA questioned?**	**Measured against (e.g. Depression**	**Age & Gender**	**Addiction rates (Prevalence)**	**Mentioned value of Internet for Academic work**	**Results**
Ade, 2018 [24]	No	None		None: 11%<br>Mild: 63%<br>Moderate: 20%<br>Severe: 6%	Yes.<br>Acknowledged in Introduction only, then no further discussion.	No usage or correlations studied.
Agah, 2016 [25]	No	None	Mean age: 21.1±3.5	None: 36%<br>Mild: 51.1%<br>Moderate: 12.3%<br>Severe: 0.6%	Yes.<br>Acknowledged in Introduction; single mention in Discussion, but not taken into account	IA correlated with:<br>• gender (M>F)<br>• marital status<br>• indigenous status.
Ahmed, 2019 [26]	No	None	Mean age: 21.1±1.5<br>Female: 73.7%<br>Male: 26.3%	None: 23.1%<br>Mild: 17.8%<br>Moderate: 48.1%<br>Severe: 11.0%	Acknowledged in Introduction.<br><br>Mentioned in Discussion, but not taken into account.	Internet used for:<br>• entertainment: 43.7%<br>• time passing: 40%<br>• social networking: 9.7%<br>• *academic: 6.6 %*<br><br>IA correlated with:<br>• academic problems<br>• time spent on the Internet.
Ahmer, 2018 [27]	No	None	Age: 21.20 ±1.67	None: 15%<br>Mild: 65.6%<br>Moderate: 18.5%<br>Severe: 0.9%	No.<br>No mention in Introduction. Academic results ignored in Discussion.	39.7% had IA and ignored academic work;<br>44.5% had AI and did not ignore academic work.

**Lead Author, Publication Date, Citation**	**Validity of IA questioned?**	**Measured against (e.g. Depression**	**Age & Gender**	**Addiction rates (Prevalence)**	**Mentioned value of Internet for Academic work**	**Results**
						IA correlated with:<br>• gender (F>M)
Alfadhul, 2018 [28]	No	None	Mean age 19.29±1.04<br>Female: 50.9%<br>Male: 49.1%	None-Mild: 44.5%<br>Moderate: 54.6%<br>Severe: 0.9%	Yes. Acknowledged in Introduction. Measured percentage of work-related time on Internet. Discussion does not discuss significance of the work-related figures on the Addiction rates.	Percentage of work-related time on Internet:<br>Normal: 67.7%<br>Addicted: 32.8%<br><br>No correlation between IA and academic performance
Ali, 2019 [29]	No	None	Mean age: 19.71±0.90	None-Mild: 46%%<br>Moderate: 53%<br>Severe: 1%<br>Males more than females.	Yes. Acknowledged in Introduction.<br>Measured academic use, and mentioned in the Discussion, but did not question the validity of the addiction rate.	54% of students used it for work-related purposes
Alpaslan, 2015 [30]	No	Hopelessness, Suicide Ideation and Hostility	Mean age: 20.5±1.8<br>Female: 55.8%<br>Male: 44.2%	None: 70.4%[a]<br>Mild: 23.3%<br>Moderate: 5.5%<br>Severe: 0.9%	No.<br><br>The correlation between AI and higher grades found in the study dismissed "probably	Positive relationship between problematic internet use and Hopelessness, Suicide Ideation and Hostility.<br><br>IA correlated with:<br>• daily use

Lead Author, Publication Date, Citation	Validity of IA questioned?	Measured against (e.g. Depression	Age & Gender	Addiction rates (Prevalence)	Mentioned value of Internet for Academic work	Results
		UCLA loneliness scale; Toronto Alexithymia Scale (TAS); Suicide Probability Scale (SPS)			related to self-report of the students"	• years of use<br>• gender (M>F)<br>• game playing<br>• UCLA<br>• TAS<br>• SPS<br>• higher grades
Al-qadasi, 2018 [8]	No (States that it is in DSM-IV)	None	Mean age: 21.6±1.98. Female: 49.8% Male: 50.2%	None: 18.5%<br>Mild: 68.7%<br>Moderate: 12.4%<br>Severe: 0.4%	Yes, acknowledged in Introduction. And not examined or discussed.	IA correlated with:<br>• more than 10 friends<br>• time on Internet<br>• higher Internet speeds
Anand, 2018 [31]	No	Psychological distress and depression	Mean age: 19.73±1.35	None: 61.8%[a]<br>Mild: 27%<br>Moderate: 10.4%<br>Severe: 0.8%	No. No mention of academic work.<br><br>The high % use of the Internet for work ignored in the Discussion.	Internet used for:<br>• *education: 8.3%*<br>• entertainment: 6.8%<br>• messaging: 2.9%<br>• *all: 81.9%*<br><br>IA correlated with:<br>• gender (M>F)<br>• amount of Internet usage

Lead Author, Publication Date, Citation	Validity of IA questioned?	Measured against (e.g. Depression	Age & Gender	Addiction rates (Prevalence)	Mentioned value of Internet for Academic work	Results
						• psychological distress<br>• inverse with age<br><br>Some balanced discussion of possible causation between psychological distress and IA.
Arya, 2018 [32]	No	None	Female: 41.55%<br>Male: 58.45%	None-Mild: 90.54%<br>Moderate: 6.21%<br>Severe: 3.23%	No. No discussion of work/education related activities.	IA correlated with:<br>• gender (M>F)<br>• rural background > urban background<br>• Internet access at home > than those without.
Asokan, 2019 [33]	No (although acknowledges not in the DSM)	None	Female: 71.9%<br>Male: 28.1%	None: 38.6%[a]<br>Mild: 63.7%<br>Moderate: 35.5%<br>Severe: 0.8%	Yes, Acknowledged in the Introduction. Value in medical practice also noted.<br><br>Mentions its academic use briefly, but does not lead to any insights about the value of "addiction".	Usage:<br>• 12.5% who use it for academic work were in the non-addicted group,<br>• 87.5% were in the addicted group.<br><br>IA correlated with:<br>• gender (M>F)<br>• time spent on Internet<br>• negative with grades

Lead Author, Publication Date, Citation	Validity of IA questioned?	Measured against (e.g. Depression	Age & Gender	Addiction rates (Prevalence)	Mentioned value of Internet for Academic work	Results
Bakarman, 2017 [34]	No	Depression (Center for epidemiological studies depression scale for children (CES-DC))	Mean age: 22.2±1.0<br>Female: 57.8%<br>Male: 42.2%[a]	IA[b]:<br>None: 50.6%[a]<br>Possible: 46.3%<br>Addicted: 3.1%	Acknowledged in the Introduction.<br><br>Impact not discussed elsewhere.	IA correlated with:<br>• gender (M > F)<br>• level of Depression<br>• inverse with year of study.
Balhara, 2015 [35]	No, (although acknowledges that it is not in the DSM)	None	Mean age: 21.23±2.66<br>Female: 50.8%<br>Male: 49.2%	None: 50.3% [a]<br>Mild: 38.7%<br>Moderate: 10.5%<br>Severe: 0.5%	No. No discussion of work-related activities.	IA correlated with:<br>• gender (M>F)<br>• average duration of daily use of the internet<br>• social networking<br>• chatting<br>• gaming<br>• shopping<br>• viewing pornography
Bansode, 2019 [36]	Yes - In the conclusion,	None	Female: 43.4%<br>Male: 56.6%	Less than average user: 6.87%<br>Average user: 67.47	Not in Introduction.<br><br>Their high education figures are referred to, but	Internet used for:<br>• social networking: 73.49%<br>• *education: 68.87%*

**Lead Author, Publication Date, Citation**	**Validity of IA questioned?**	**Measured against (e.g. Depression**	**Age & Gender**	**Addiction rates (Prevalence)**	**Mentioned value of Internet for Academic work**	**Results**
	mention that it is debated and not in DSM.			Over-users: 12.05<br>Addicts: 6.38	do not impact on their interpretation of IA.	• downloading: 62.65%<br>• gaming: 12.05%<br><br>IA correlated with:<br>• gender (M>F)
Berner, 2014) [37]	Yes, acknowledges that IA is not firmly established	Depression (Goldberg's General Health Questionnaire (GHQ-12))	Mean age: 20.78±1.79<br>Male: 58.3%	IAT:<br>Non-problematic (<50 on score):88.3%[a]<br>Problematic (>=50 on score):11.7%	No.<br><br>No mention of academic work in the paper.	IA correlated with:<br>• GHQ-12
Boonvisudhi, 2017 [38]	No, although mentions that there is no consensus on the clinical definition.	Depression (The Patient Health Questionnaire (PHQ-9))		Mean IA score = 3.27;<br><br>24.4% had possible IA.	No.	IA correlated with:<br>• depression<br>• academic problems<br>• health problems

Lead Author, Publication Date, Citation	Validity of IA questioned?	Measured against (e.g. Depression	Age & Gender	Addiction rates (Prevalence)	Mentioned value of Internet for Academic work	Results
Capetillo-Ventura, 2015 [39]	No	Mental Health General Health Questionnaire, and the Zuckerman-Kuhlman Personality Questionnaire III (ZKPQ)	Mean age: 21.24±3.046<br>Female: 46.2%<br>Male: 53.8%	IAT<br>Mean score: 19.72<br><br>IA:<br>had complete control over its use (20-39): 91.8%<br>frequent problems: (40-69): 8%<br>significant problems: (>70): 0.2%.	Yes<br>Acknowledged in Introduction.<br><br>The extremely high use for education and research ignored in the discussion. It is mentioned in the conclusion, but does not impact of the interpretation of the results.	Internet main usage for:<br>• social networks: 43.86%<br>• *academic and research activities: 32.95%*<br>• entertainment/leisure in 23.18%.<br><br>IA correlated with:<br>• gender (M>F)
Chakraborti, 2016 [40]	No	Resilience scale and student stress scale (SSS).	Mean age: 20.41±1.64<br>Female: 35.7%<br>Male: 64.3%	IA:<br>Moderate users: 80.6%<br><br>19.4% were problem users.	No.<br><br>Mentioned in the Discussion (that is among the highest uses of the Internet), but not taken into account with the interpretation.	Internet used for:<br>• social networking: 76.5%<br>• *academic activity and research: 74.5%*<br><br>IA correlated with:<br>• gender (M>F)<br>• SSS total score<br>• total number of stressful life events

Lead Author, Publication Date, Citation	Validity of IA questioned?	Measured against (e.g. Depression	Age & Gender	Addiction rates (Prevalence)	Mentioned value of Internet for Academic work	Results
						• negatively correlated with resilience
Chathoth, 2013 [41]<br><br>Chathoth, 2014 [42]	No	None	Mean age: 18.49± 0.71<br><br>Female: 62.2%<br>Male: 37.8%	IA:<br>Normal: 23.33%<br>Mild: 57.77%<br>Moderate: 18.88%<br>Severe: 0%	No<br><br>Brief mention in the Discussion, but not taken into account on the interpretation of the results.	Internet used for:<br>• social networking: 97.8%<br>• e-mail: 87.8%<br>• *education: 82.2%*<br>• entertainment: 82.2%<br><br>IA correlated with:<br>• sleep impairment<br>• excessive daytime sleepiness<br>• environmental stressors
Chaudhari, 2015 [43]	No, but acknowledges not in DSM.	None	Mean age: 19.90±1.37<br>Female: 56.74%<br>Male: 43.26%	IA:<br>None: 41.13%<br>Mild: 51.42%<br>Moderate: 7.45%	Acknowledged in Introduction. Although, give a list of 5 aspects that make students more vulnerable to AI, and education is not mentioned.<br><br>No mention of academic work in the Discussion.	IA correlated with:<br>• gender (M>F)<br>• staying in private accommodation<br>• lesser age of first internet use<br>• using mobile for internet access<br>• higher expenditure on internet<br>• staying online for longer time

**Lead Author, Publication Date, Citation**	**Validity of IA questioned?**	**Measured against (e.g. Depression**	**Age & Gender**	**Addiction rates (Prevalence)**	**Mentioned value of Internet for Academic work**	**Results**
						• using internet for social networking, online videos<br>• watching website with sexual content
Chaudhuri, 2019 [44]	No	The Duke Health Profile		IA:<br>None: 25.87<br>Mild: 58.70<br>Moderate: 15.42	No.<br><br>High usage for educational purposes mentioned in Discussion, but does not affect the interpretation of the IA.<br><br>Recommendations include avoid internet at night, during classes and lectures, in spite of the 83% usage for education.	Internet used for:<br>• *education: 83.0%*<br>• social networking: 76%<br><br>IA correlated with:<br>• Anxiety score<br>• Depression score<br><br>IA negatively correlated with:<br>• Physical health score<br>• Mental health score<br>• Perceived health score
Ching, 2017 [45]<br><br>Mooi, 2019 [46]	No	Depression	Mean age: 21.60±1.50<br>Female: 63.4%<br>Males: 36.6%	Addicted: 36.9%	Yes.<br>Acknowledged in Introduction as important for medical students for “literature searches and searching for relevant medical information.”	*51.6% use Internet for education purposes.*<br><br>IA correlated with:<br>• gender (M>F)<br>• duration of internet use<br>• DASS-21 Score

Lead Author, Publication Date, Citation	Validity of IA questioned?	Measured against (e.g. Depression	Age & Gender	Addiction rates (Prevalence)	Mentioned value of Internet for Academic work	Results
					The education usage is not mentioned in the Discussion, and does not influence the interpretation.	
Damor, 2018 [47]	No	None	Female: 48.6%<br>Male: 51.4%	IA:<br>Less than average users: 23.4%<br>Average online users: 59.1%<br>Possible addict: 17.2%<br>Addict: 0.3%	Yes, Acknowledged in Introduction.<br><br>Mentioned in the Discussion, but no impact on the interpretation of results.	Internet used for:<br>• social media: 65.2%<br>• *academic literature search: 19.4%*
Daripelly, 2017 [48]	No	General health questionnaire (GHQ - 12)	Female: 49.4%<br>Male: 50.6%	IA (Medical students only):<br>Non-Addict: 46.3%<br>Potential Addict: 27.5%<br>Internet Addict: 21.6%	No mention in the Introduction.<br><br>The results show that, overall, and for the “potential addicts” the most common use of the Internet is for research purposes. This is ignored in the Discussion and the interpretation of the results.	Internet most commonly used for:<br>• *research: 35.1%*<br>• socialization: 34.7%<br>• entertainment: 24.6%<br>• others: 5.5%<br><br>IA correlated with<br>• gender (M>F)<br>• parents’ level of education<br>• more hours of daily internet use

Lead Author, Publication Date, Citation	Validity of IA questioned?	Measured against (e.g. Depression	Age & Gender	Addiction rates (Prevalence)	Mentioned value of Internet for Academic work	Results
						• use the internet more frequently each week<br>• use the internet for socialization and entertainment<br>• GHQ scores
Duică, 2017 [49]	No	None		IA:<br>No/Mild: 91.5%<br>Moderate: 8.2%<br>Severe: 0.4%	Acknowledges some positive contribution of Internet to work-related activities.	Found high use of the Internet for work, and "those with internet addiction are the ones that use the internet as information source for professional development."<br><br>IA negatively correlated with:<br>• watching medical videos before exams<br>• internet socialization (including topics from professional training)
Fatehi, 2016 [50]	No	WHOQOL-BREF	Mean age: 22.57±1.24<br>Female: 55.7% [a]<br>Male: 44.3%	IA:<br>Normal (<50): 83.9%<br>Addicted (>= 50): 16.1%	No.<br><br>No mention of the Mentions Internet as a tool for work.	GPA lower in addicted group.<br><br>IA negatively correlated with:

**Lead Author, Publication Date, Citation**	**Validity of IA questioned?**	**Measured against (e.g. Depression**	**Age & Gender**	**Addiction rates (Prevalence)**	**Mentioned value of Internet for Academic work**	**Results**
						• average of QOL domains
Gaddala, 2017 [51]	No	None	Female: 64%<br>Male: 36%	IA:<br>None: 24%<br>Mild: 61%<br>Moderate: 14%<br>Severe: 1%	Acknowledged in Introduction.<br><br>The fact that 100% of the student use the Internet for educational purposes is ignored in the discussion.	*100% of the students use the Internet for educational purposes.*<br><br>IA correlated with:<br>• Gender (M>F)
Gedam, 2016 [52]	No. Acknowledge the work by [120], but still proceeded.	Depression Mental health inventory	Mean age: 19.71±0.97<br><br>Female: 63.5%<br>Male: 36.5%	IA:<br>None/Mild: 81.5%<br>Moderate: 17.3%<br>Severe: 1.2%	Acknowledged in the Introduction.<br><br>High usage of Internet for educational work is not mentioned in the discussion.	Internet used for:<br>• social networking: 31.86%<br>• *education: 28.92%*<br>• games: 17.91%<br>• recreational: 17.32%<br>• others: 3.96%<br><br>IA correlated with:<br>• gender (M>F)<br>• computer ownership<br>• purpose of internet use
Gedam, 2016 [53]	No	Anxiety, depression, distress	Mean age: 19.57±1.52<br>Female: 62.8%	IA<br>None/Mild: 76.7%<br>Moderate: 21.0%<br>Severe: 2.3%	Yes.<br>Acknowledged in Introduction.<br>High usage of Internet for educational work is not	Internet used for:<br>• social networking: 31.08%<br>• *education: 28.87%*<br>• recreational: 18.26%<br>• games: 16.69%

Lead Author, Publication Date, Citation	Validity of IA questioned?	Measured against (e.g. Depression	Age & Gender	Addiction rates (Prevalence)	Mentioned value of Internet for Academic work	Results
			Males: 37.2%		mentioned in the discussion.	• others: 5.07%<br><br>IA correlated with:<br>• gender: (M>F)<br>• anxiety<br>• depression<br>• loss of emotional / behavioral control<br>• psychological distress<br><br>IA negatively correlated with:<br>• life satisfaction<br>• psychological well-being
George, 2019 [9]	No. And says that the DSM does include internet addiction disorder.	None	Mean age: 21.3±1.25	IA<br>None: 29% [a]<br>Mild: 54%<br>Moderate: 17%	Acknowledged in Introduction.<br><br>Did not ask the students what they did, and so this aspect not covered in the Discussion.	IA correlated with:<br>• gender (M>F)<br>• mobile/tablet usage<br>• eye strain<br>• time to initiate sleep<br>• frequency of sleep trouble<br>• hours spent on internet<br>• money spent<br>• presence of backache<br>• appetite change

Lead Author, Publication Date, Citation	Validity of IA questioned?	Measured against (e.g. Depression	Age & Gender	Addiction rates (Prevalence)	Mentioned value of Internet for Academic work	Results
Ghamari, 2011 [54]	No	None	Mean age: 21[a]<br>Female: 64.9%<br>Male: 35.1%[a]	IA:<br>Mean score: 32.74±14.52<br>Moderate: 8%<br>Severe: 2.8%	Acknowledged in Introduction.<br><br>The fact that the students used the Internet mostly for work is repeated in the Discussion, but does not influence the researchers' interpretation of their view on addiction.	Internet used for:<br>• *research and scientific surveys: 48.4%*<br>• computer games and hobbies: 20.5%<br>• checking email: 9.8%<br>• political and social news: 6.5%<br>• chat rooms: 5.6%<br><br>IA correlated with:<br>• gender (M>F)<br>• marital status (M>S)<br>• father's job<br>• knowledge about computer and internet<br>• educational level.
Ghanate, 2019 [55]	No	Becks Depression scale; Beck's Anxiety Inventory	Female: 53.14%<br>Male: 46.8%	IA:<br>Normal: 80.9%<br>Moderate: 17.4%<br>Severe: 1.7%	Acknowledged in Introduction.<br><br>In spite of these work figures, this is not mentioned in the Discussion, nor does it	Internet used for:<br>• entertainment: 81%<br>• *academic work: 36.1%*<br>• social friendships: 35%<br>• loneliness: 11.4%<br><br>IA correlated with:<br>• Anxiety

Lead Author, Publication Date, Citation	Validity of IA questioned?	Measured against (e.g. Depression	Age & Gender	Addiction rates (Prevalence)	Mentioned value of Internet for Academic work	Results
					impact on the interpretation of the results	• Depression
Ghosh, 2018 [56]	No	None	Female: 44.5%<br>Male: 55.5%	IA:<br>None: 44.5%<br>Mild: 34.8%<br>Moderate: 19.4%<br>Severe: 1.3%	Mentioned in the Abstract, but not in the Paper's Introduction.<br><br>Discussion talks about the importance of medical persons using the Internet to stay up to date, does not affect the interpretation of these results, and does not addressed the anomaly that students appear to not access their learning materials online.	Internet used for:<br>• online chatting: 78.7%<br>• social networking: 61.9%<br><br>IA correlated with:<br>• gender (M>F)<br>• duration of daily usage<br>• amount of money spent for Internet usage

Lead Author, Publication Date, Citation	Validity of IA questioned?	Measured against (e.g. Depression	Age & Gender	Addiction rates (Prevalence)	Mentioned value of Internet for Academic work	Results
Güzel, 2018 [57]	No	None	Age range from 18-26.<br>Female: 54.13%<br>Male: 45.87%	[Did not use Young's scale]	Acknowledged in Introduction.<br><br>In spite of the high usage for educational work, no reference is made in the Discussion, and it does not affect their interpretation.	Internet used for:<br>• communicational purposes: 94.5%<br>• *educational purposes: 68.9%*<br><br>IA correlated with:<br>• some physical complaints<br>• insomnia<br>• time spent on the internet
Hajare, 2017 [58]	No	None	Age 21.2[a]<br><br>Female: 42.54%<br>Male: 57.4%	IA:<br>None: 33.58%<br>Mild: 23.38%<br>Moderate: 39.55%<br>Severe: 3.48%	No mention in Introduction, or anywhere else in the paper.	IA correlated with:<br>• both the parents working<br>• had internet connections at their home or hostel room<br>• using internet for more than 5 years<br>• used internet for more than 28 hrs/week<br><br>No correlation with age or gender was found.

Lead Author, Publication Date, Citation	Validity of IA questioned?	Measured against (e.g. Depression	Age & Gender	Addiction rates (Prevalence)	Mentioned value of Internet for Academic work	Results
Hamissi, 2013 [59]	No	Emotional Intelligence	Mean age: 23.26±3.36<br>Female: 31.8%[a]<br>Male: 68.2%	IA:<br>Normal: 38.3%<br>Mild: 43.8%<br>Moderate: 15.9%<br>Severe: 2%	No<br><br>No mention of education influence at all, in spite of the high usage of the Internet for Education, and in spite of the fact that the PhD students use the Internet more.	Internet used for:<br>• *read articles: 33.8%*<br>• download music/images: 7.7%<br>• chat: 1.2%<br>• games: 1.5%<br>• not at all: 1%<br><br>Usage of Computer:<br>• games: 43.8%<br>• *education: 25.0%*<br>• communication: 15.4%<br>• entertainment: 5.1%<br>• not at all: 1%<br><br>IA correlated with:<br>• gender (M>F)<br>• education grade: PhD having the highest.<br>• Internet usage<br>• history of depression
Haque, 2016 [60]	No	None	Mean age: 21.99±1.535	Both male and female suffered from mild IA	Acknowledged in Introduction.	Internet used for:<br>• social networking: 56.2%<br>• entertainment: 39.7%

Lead Author, Publication Date, Citation	Validity of IA questioned?	Measured against (e.g. Depression	Age & Gender	Addiction rates (Prevalence)	Mentioned value of Internet for Academic work	Results
			IA Mean Scores:<br>Female: 41.4±13.05<br>Males: 44.9±14.05		Work usage in Results ignored in the Discussion. No reasons given.	• *educational: 34.9%*<br>• *research: 12.3%*<br>• email: 12.3%<br>• conferences: 3%<br><br>IA negative correlated with:<br>• year of study<br>• age<br><br>IA did not correlate with:<br>• usage<br>• work<br>• gender
Hayat, 2020 [61]	No	Procrastination	Female: 57.1%<br>Male: 42.9%	IA:<br>Severe: 3.43%	Yes	IA correlated with:<br>• gender (M>F)<br>• living in dorms<br>• procrastination
Hussain, 2018 [62]	No	None	Female: 56.7%<br>Male: 43.3%	IA Prevalence: 41.7%.	No Acknowledged in Introduction.<br><br>Academic figures not mentioned in Discussion.	Internet mainly used for:<br>• entertainment: 38.3%[a]<br>• social: 33.3%<br>• *research/academics: 15.8%*<br>• other: 12.5%

**Lead Author, Publication Date, Citation**	**Validity of IA questioned?**	**Measured against (e.g. Depression**	**Age & Gender**	**Addiction rates (Prevalence)**	**Mentioned value of Internet for Academic work**	**Results**
						IA correlated with:<br>• year of study (2nd > 3rd)<br>• Type B personality > Type A
Ja'ffar, 2019 [63]	No	None	Female: 59.32%[a]<br>Male: 40.68%[a]	IA:<br>None: 10.6%<br>Mild: 40.3%<br>Moderate: 45.6%<br>Severe: 3.4%	Acknowledged in Introduction.<br><br>Mentioned only<br><br>No significant association between IA and educational use, but the fact that 56% of the students use it for educational purposes ignored in the interpretation.	Internet used for:<br>• social network: 89.7<br>• education: 56.3%<br>• gaming: 30.4<br>• e-mail: 20.2<br><br>IA Correlated with:<br>• hours spent online per day<br><br>No correlations with: gender, parents' marital status, academic grade, residence and parents' level of education, years of internet use, gaming.
Jain, 2018 [64]	No	None	Female: 49.4%<br>Male: 50.6%	IA:<br>Normal: 6.7%<br>Mild: 38.7%<br>Moderate: 49.3%<br>Severe: 5.3%	Acknowledged in Introduction.<br><br>High use for Academic work ignored in the interpretation.	Internet used for:<br>• chatting: 100%<br>• YouTube: 100%<br>• online friendship/ relationship: 94%<br>• *academic work: 69.3%*

Lead Author, Publication Date, Citation	Validity of IA questioned?	Measured against (e.g. Depression	Age & Gender	Addiction rates (Prevalence)	Mentioned value of Internet for Academic work	Results
						• movies: 62.7%<br>• shopping: 58%<br>• games: 41.3%<br>• pornography: 14.7%<br><br>IA correlated with:<br>• staying in hostel and living single<br>• usage of smart phone<br>• preferring online shopping<br>• preferring online studying.<br><br>No correlation with gender.
Javaeed, 2019 [65]	No, but mentions that it is not in DSM.	DASS21 for depressioNone	Female: 64.3%[a]<br>Male: 35.7%	IA:<br>None: 3.3.%<br>Mild: 44.3%<br>Moderate: 51.0%<br>Severe: 1.4%	Acknowledged in the Introduction.<br><br>No further mention of Internet for work-related activities.	IA Correlated with:<br>• depression<br>• stress
Javaeed, 2020) [66]	No	None	Female: 59.2%<br>Male: 40.8%[a]	IA:<br>None: 0.9%<br>Mild: 12.0%<br>Moderate: 58.9%<br>Severe: 28.2%	Yes, in Introduction. No further mention of value to work.	IA correlated with:<br>• year of study (third year students scoring the highest)

**Lead Author, Publication Date, Citation**	**Validity of IA questioned?**	**Measured against (e.g. Depression**	**Age & Gender**	**Addiction rates (Prevalence)**	**Mentioned value of Internet for Academic work**	**Results**
						IA negatively correlated with:<br>• academic performance<br><br>IA did not correlate with age or gender.
Kannan, 2019 [67]	No	Auditory reaction time-ART, visual reaction time VRT Heart Rate Variability (HRV)	Female: 36.8%<br>Male: 63.2%	IA:<br>Not: 82.6%[a]<br>Addict: 17.4%	No mention of it in the paper.	Auditory reaction time was significantly prolonged in the internet addicts compared to the non-addicts.<br><br>IA correlated with:<br>• gender (M>F)
Kapadia, 2015 [68]	No	None	Mean age: 21.75±2.96<br>Female: 37%<br>Male: 63%	IA:<br>Normal: 17.5%<br>Mild: 62.5%<br>Moderate: 20%<br>Severe: 0%	No, Not mentioned in Introduction.<br><br>Results of high use for study repeated in the Discussion, but no	Internet used for:<br>• entertainment: 83.5%<br>• *study: 80.5%*<br>• chatting: 80.5%<br>• email: 61.5%<br>• online shopping: 61%<br>• *research: 49%*

Lead Author, Publication Date, Citation	Validity of IA questioned?	Measured against (e.g. Depression	Age & Gender	Addiction rates (Prevalence)	Mentioned value of Internet for Academic work	Results
					significance of that figure made.	
Khan, 2016 [69]<br>Khan, 2017 [70]	No	Academic performance	Female: 45.7%<br>Male: 54.3%	IA:<br>Normal: 83.2%<br>Moderate: 16.1%<br>Severe: 0.6%	Acknowledged in Introduction of both papers.<br><br>No mention of it in the Discussion.	IA correlated negatively with:<br>• academic performance.<br>• physical activity
Komleh, 2015 [71]	No	academic achievement (GPA)	Female: 58%<br>Male: 42%	IA<br>15.2% had IA (taken as > 44 on the scale).	No mention in Introduction.<br><br>No measure of activities, although the discussion does note the impact on critical thinking and lower failure rate, but still does not reinterpret the "addiction" rates.	IA correlated with :<br>• time spent on the Internet<br><br>IA inversely correlated with<br>• failure of credits (higher IA, fewer failures).<br><br>No correlation with gender, GPA
Kootesh, 2016 [10]	No, and indicates that it is in DSM-5	Pittsburgh Sleep Quality Inventory (PSQI)<br>Mental Health	Female: 52%<br>Male: 48%	IA Mean: (37.17±13.40)	No mention in the Introduction or Discussion.	IA negative correlated with:<br>• sleep quality<br>• mental health

**Lead Author, Publication Date, Citation**	**Validity of IA questioned?**	**Measured against (e.g. Depression**	**Age & Gender**	**Addiction rates (Prevalence)**	**Mentioned value of Internet for Academic work**	**Results**
		Checklist (MHC)				
Kumar, 2017 [72]	No	None	Mean age: 20.67±1.08<br>Female/Male : 50/50	IA<br>No addiction: 60%<br>Mild: 30%<br>Moderate: 10%	Acknowledged in Introduction.<br><br>No mention of work in the Discussion, so the 61% using it for study purposes ignored.	Internet used for:<br>• entertainment: 62.7%<br>• *study: 61.3%*<br>• keeping in touch with friends and family: 61.3%<br><br>IA correlated with:<br>• gender (M>F)<br>• hours of internet usage per day<br>• expenditure on Internet
Kundu, 2017 [73]	No	Becks depression scale	Mean age: 20.15±1.89<br>Female: 75.38%<br>Male: 24.62%	IA:<br>None: 17.6%<br>Mild: 59.23%<br>Severe: 23.08%	Introduction: Mentions use of internet by health professionals for work, but not about students for studying.<br><br>Makes no reference to the high academic percentage in the results.	Internet used for:<br>• social media: 73.08%<br>• downloading media files: 65.38%<br>• academic purposes: 63.08%<br>• shopping: 51.54%<br><br>IA correlated with:<br>• gender (M>F)

Lead Author, Publication Date, Citation	Validity of IA questioned?	Measured against (e.g. Depression	Age & Gender	Addiction rates (Prevalence)	Mentioned value of Internet for Academic work	Results
						IA negatively correlated with:<br>• depression
Latt, 2017 [74]	No	Islamic principles	Female: 64.1%<br>Male: 35.9%	IA:<br>Normal: 5.8%, 48.5% mild, 44.7 moderate, 1% severe	Acknowledged in the Introduction.<br><br>Some of the academic-related benefits of the Internet are explored in the Discussion, but does not impact on interpretation of the addiction rates. However, it gives a more balanced view than most of the other papers.	
Liu, 2009 [75]	No	Academic performance	Female: 48.9%[a]<br>Male: 51.1%[a]	Prevalence of IAD:<br>Female: 11.6%<br>Male: 20.6%	Acknowledged in Introduction<br><br>The high proportion of work-related activity is noted in the Discussion, and appears to moderate their authors fears of addiction.	"Frequent" use of Internet:<br>• email: 51.4%<br>• searching for information: 34.4%<br>• chatting: 29.3%<br>• news: 25.0%<br>• entertainment news: 24.1%<br>• music: 23.0%<br>• films: 20.7%

Lead Author, Publication Date, Citation	Validity of IA questioned?	Measured against (e.g. Depression	Age & Gender	Addiction rates (Prevalence)	Mentioned value of Internet for Academic work	Results
						IA correlated with:<br>• gender (M>F)<br><br>IA negatively correlated with:<br>• academic performance.
Loredo e Silva, 2018 [76]	No	Biggs' Study Process Questionnaire (R-SPQ-2F):	Mean age: 22.11±3.11<br>Female: 55.4%<br>Male: 44.6%[a]	IA Mean score: 46.27 (SD:12.12);<br>Non-problematic: 31.8%<br>Problematic: 68.2% (Frequent= 64.6%; significant= 3.6%)	No. Mentions educational value of smartphones, but not Internet (it might be implied)	Had medical apps: 67.8% (of the clerkship students, this rose to 93.4%)<br>Use phone apps for learning: some 30-50% of the students.<br><br>IA correlated with:<br>• surface learning approaches<br>• lower scores in deep learning approaches
Madhusudan, 2018 [77]	No, but mentions that it is not in	None	Mean age: 21.66±1.59<br>Female: 65.6%<br>Male: 34.4%	IA:<br>None: 5.5%<br>Mild: 60.8%<br>Moderate: 31.3%<br>Severe: 2.5%	Acknowledged in the Introduction.<br><br>No mention of the 20% used "to gain knowledge"	Internet used for:<br>• social networking: 55.93%<br>• communication: 21.81%<br>• *gain knowledge: 20.58%*<br>• others: 1.63%

Lead Author, Publication Date, Citation	Validity of IA questioned?	Measured against (e.g. Depression	Age & Gender	Addiction rates (Prevalence)	Mentioned value of Internet for Academic work	Results
	the DSM.				and any modification of addiction. Also, the Communication is ill-defined, and may be work-related or not.	IA correlated with:<br>• gender (M>F)<br>• phase of degree (Final>others)<br>• place of origin (urban>rural)<br><br>IA negatively correlated with:<br>• previous marks obtained
Mallya, 2019 [78]	No	Depression, anxiety, and stress scale 21; Heart rate and BP components such as systolic and	Age: 19.56[a]<br>Female: 61%<br>Male: 39%	IA rate: 44%<br>(Female: 52%<br>Male: 31%)	Acknowledged in the Introduction.<br><br>No mention of work in the Discussion.	Internet used mainly for:<br>• social media: 85%<br>• gaming: 10%<br>• academic: 4%<br>• shopping: 2%<br><br>IA correlated with:<br>• stress<br>• depression

Lead Author, Publication Date, Citation	Validity of IA questioned?	Measured against (e.g. Depression	Age & Gender	Addiction rates (Prevalence)	Mentioned value of Internet for Academic work	Results
		diastolic BP				
Malviya, 2014 [79]	No	None	Age range: 21-25.<br>Female: 32.2%<br>Male: 67.8%	IA:<br>None: 7.4%<br>Average: 64.5%<br>Moderate: 18.6%<br>Severe: 9.5%	No mention of work in the Introduction, or anywhere else in the paper.	No correlation with gender.
Mohammadbeigi, 2016 [80]	No	Self-Rated Health	Mean age: 21.7±2.9<br>Female: 84.3%<br>Male: 15.7%[a]	28.7% were addicted; mean score: 35.22±12.1.	Yes, in the Introduction. The high percentage usage for work-related activities is not raised in the Discussion.	64.2% use it for "scientific search" and 37.0% use it for "Academic research".<br><br>IA correlated with:<br>• Gender (F>M)<br>• Entertainment<br>• Chat rooms<br>• Have email<br>• Use of Internet<br><br>IA negatively correlated with:<br>• Self-reported Health<br>• academic performance
Mostafa, 2019 [81]	No	None	Mean age: 21.78±1.48<br>Female: 74.0%	IA:<br>Mild: 63.69%<br>Moderate: 35.43%<br>Severe: 1.18%	Discussed in some detail in the Introduction.	Internet used for:<br>• social networking: 86.61%<br>• *education: 66.53%*

Lead Author, Publication Date, Citation	Validity of IA questioned?	Measured against (e.g. Depression	Age & Gender	Addiction rates (Prevalence)	Mentioned value of Internet for Academic work	Results
			Male: 26.0%		Mention of it in the Discussion but no interpretation impact on the Results	• entertainment: 61.81%<br>• e-mail communication: 21.25%<br>• *research: 9.05%*<br>• net meeting: 5.51%<br>• others: 1.57%
Mukherjee, 2020 [82]	No	Insomnia Severity Index; Rosenberg Self Esteem Scale (RSES)	Mean age: 20.0[a]<br><br>Female: 48.7%<br>Male: 51.3%	Average IA score: 51.76 ± 16.96<br><br>IA:<br>"Average" (20-39): 30.0%<br>"over-users" (40-69): 50.7%<br>"internet addicts" (70-100): 19.3%	Acknowledged in the Introduction.<br><br>No mention of work in the paper.	IA correlated with:<br>• gender (M>F)<br><br>IA correlated negatively with:<br>• self-esteem
Nagori, 2016 [83]	No	Beck's Anxiety Inventory (BAI); World health organization quality of life	Female: 53.33%<br>Male: 46.67%	IA:<br>Less than average: 34.3%<br>Average: 56.4%<br>Moderate: 8.4%<br>Addict: 0.9%	Acknowledged in Introduction.<br><br>No mention of the high use of Internet for academic purposes in the Discussion.	Internet mainly used for:<br>• social networking: 81.5%<br>• *education: 81.3%*<br>• entertainment: 72.2%<br><br>IA correlated with:<br>• gender (M>F)

Lead Author, Publication Date, Citation	Validity of IA questioned?	Measured against (e.g. Depression	Age & Gender	Addiction rates (Prevalence)	Mentioned value of Internet for Academic work	Results
		assessment scale (WHOQOL-BREF).				
Nath, 2016 [84]	No, although notes that Goldberg introduced it as satire, and that it is not in DSM 5.	None	Mean age: 22.51±2.91<br>Female: 39.4%<br>Male: 60.6%	IA:<br>Less than average: 9.04%<br>Average: 44.15%<br>Possible addict: 46.28<br>Addict: 0.53	Acknowledged in Introduction.<br><br>No mention of academic work in Discussion.	Internet used for:<br>• social networking: 81.5%<br>• *education: 81.3%*<br>• entertainment: 72.2%<br>• social networking: 67.0%<br>• academic content: 13.3%<br>• downloadable media: 9.6%<br>• social media: 8.5%<br>• pornography: 1.6%.<br><br>IA correlated with:<br>• gender (M>F)<br>• preferred to always stay online<br>• exposed to the Internet for 6 years or more<br>• forming online relationships

Lead Author, Publication Date, Citation	Validity of IA questioned?	Measured against (e.g. Depression	Age & Gender	Addiction rates (Prevalence)	Mentioned value of Internet for Academic work	Results
						• poor performance at college<br>• feelings of anxiety and depression
Öztürk Kaygusuz, 2019 [85]	No, although says it is not in the DSM.	None	Mean age: 20.47±1.88<br>Female: 52.6%<br>Male: 47.4%[a]	IAT Mean Score: 29.92±16.33<br><br>Not Addicts: 77.5%<br>Probable Addicts: 19.4%<br>Addicts: 3%	Not in introduction.<br><br>Results that show correlation to work noted in the Discussion, yet has no impact on the interpretation of the results.	IA correlated with:<br>• time on Internet<br>• *internet use for information search*<br>• *course-training-research*<br>• video conversation<br>• suffering from sleeplessness
Patel, 2018 [86]	No	None	Mean age: 19.4.<br>Female: 41.72%<br>Male: 58.28%	Possible Addicts: 74.82%<br>Severe: 16.55%<br>Mild: 8.63%	In the Introduction, mentions that it is useful for “knowledge sharing”<br><br>Academic use results ignored in the Discussion	Academic purposes bundled with gaming, social media, so it not possible to extract.
Patil, 2017 [87]	No	None	Mean age: 20<br>Female: 43.65%<br>Male: 56.35%	IA:<br>Average: 61.47%<br>Possible: 34.83%<br>Addicts: 3.68%	Acknowledged in Introduction.<br><br>No mention in paper of use of Internet for academic work	IA correlated with:<br>• gender (M>F)

Lead Author, Publication Date, Citation	Validity of IA questioned?	Measured against (e.g. Depression	Age & Gender	Addiction rates (Prevalence)	Mentioned value of Internet for Academic work	Results
Pramanik, 2012 [88]	No	None	Female: 50%<br>Male: 50%	IA:<br>Mild: 40%<br>Moderate: 41.53%<br>Severe: 3.05%<br>None: 15.42%[a]	Acknowledged in Introduction.<br><br>No measure of educational usage; no discussion of educational usage in the paper.	
Qadir, 2018 [89]	No	None	Mean age: 22.02[a]<br>Females: 50%<br>Males: 50%	IA:<br>Normal: 53%<br>Problematic: 45%<br>Addict: 2%	No mention in the Introduction.<br><br>The fact that 20% used it mainly for study does not influence the interpretation.	Internet mainly used for:<br>• entertainment: 53%<br>• study: 20%<br>• information: 15%<br>• communication: 9%<br>• gaming: 2%<br>• shopping: 1%
Radeef, 2018 [90]	No, although noted that it is not included in DSM-5. Compared to Internet	Depression Anxiety, Stress Scale (DASS-21)	Mean age: 22.87 years<br>Female: 30.6%<br>Male: 69.4%[a]	22.8% were IA	Acknowledged in the Introduction.<br><br>No further mention of educational work in their paper.	IA correlated with:<br>• depression<br>• anxiety symptoms<br><br>IA negatively correlated with:<br>• social interaction (inverse)

**Lead Author, Publication Date, Citation**	**Validity of IA questioned?**	**Measured against (e.g. Depression**	**Age & Gender**	**Addiction rates (Prevalence)**	**Mentioned value of Internet for Academic work**	**Results**
	gaming disorder					
Rebello, 2017 [91]	No	None	Female: 42.6%[a]<br>Male: 57.4%[a]	CUI prevalence: 11.21%	Acknowledged in Introduction.<br><br>Academic results referred to in Discussion, but no implications on the CUI of this discussed.	Internet used for:<br>• *Studies: 96.1%*<br>• Get connected with people: 90.6%<br><br>Felt:<br>• Technology should be utilized: 96.1%<br>• Usage can be restricted: 81.3%<br>• Internet usage as distraction: 58.9%<br>• Hinder academics: 47.2%<br><br>CUI correlated with:<br>• mobile usage duration<br>• Internet usage duration<br>• maximum usage time<br>• gender (M>F)
Rustam, 2017 [92]	No, although mentions that it is	None	Age range: 18-25.<br>Female: 53%<br>Males: 47%	IA:<br>Normal: 27%<br>Mild: 45.5%<br>Moderate: 27%	No mention in the Introduction.	Only IA results reported.

Lead Author, Publication Date, Citation	Validity of IA questioned?	Measured against (e.g. Depression	Age & Gender	Addiction rates (Prevalence)	Mentioned value of Internet for Academic work	Results
	not in DSM.			Severe: 0.5%	No mention of work or academics in the paper.	
Sahraian, 2016 [93]	No	Personality (NEO-FFI)	Mean age: 21.48±2.59<br>Female: 61%<br>Male: 39%	IA<br>Normal: 45.3%<br>Mild: 51.4%<br>Moderate: 2.9%<br>Severe: 0.4%	Yes, mention academic work on Internet.<br><br>Academic work, including the high percentage in these results, ignored in the Discussion	Internet used for:<br>• *scientific research: 64.0%*<br>• social web: 63.3%<br>• e-mail: 59%<br>• non -scientific research: 57%<br>• music: 53.2%<br>• film: 41%<br>• software: 41%<br>• chat: 30.2%<br>• news: 39.2%<br>• gaming: 17.6%<br><br>IA correlated with:<br>• neuroticism<br>• gender (M>F)<br>• live with family > Residence<br>• single > married<br><br>IA negatively correlated with:<br>• extraversion

Lead Author, Publication Date, Citation	Validity of IA questioned?	Measured against (e.g. Depression	Age & Gender	Addiction rates (Prevalence)	Mentioned value of Internet for Academic work	Results
						• agreeableness<br>• conscientiousness
Saied, 2016) [94]	No	None	Mean age: 22.08±1.15<br><br>Female: 56.56%[a]<br>Male: 43.44%[a]	IA:<br>Average: 64.1%<br>Potentially addicted: 33.2%<br>Addicted: 2.7%	No. The high usage figure of the Internet for academic purposes ignored in the Discussion.	Internet used for[a]:<br>• Facebook: 92.0%<br>• *information & research: 80.0%*<br>• other social media: 77.0%<br>• entertainment: 76.9%<br>• *studying: 72.9%*<br><br>Of those who used it for Facebook, 79.1% used it for educational purposes.<br><br>IA correlated negatively with:<br>• academic grades<br><br>Adverse effects: eye irritation, followed by headache, then back pain
Saini, 2016 [95]	No	Personality Traits Goldberg's Big-Five	Female: 36.6%<br>Male: 63.4%	Mean IA score: 33.94 (SD 13.592)<br>No other IA figures given.	No mentioned in the Introduction.<br><br>No mention of work or academics in paper.	IA correlated with:<br>• higher neuroticism<br>• less extroversion

Lead Author, Publication Date, Citation	Validity of IA questioned?	Measured against (e.g. Depression	Age & Gender	Addiction rates (Prevalence)	Mentioned value of Internet for Academic work	Results
		factor test				
Salehi, 2014 [96]	No	None	Mean age: 21.79±2.42<br>Female: 61.1%<br>Male: 38.9%	Not addicted: 92.7%<br>At risk: 2.1%<br>Addicted 5.2%	No mention in the Introduction.<br><br>Although scientific research result referred to in the Discussion this did not impact on the interpretation of data.	Internet used for<br>• email: 54%<br>• *scientific research: 39.9%*<br>• communicating with friends and family: 35%<br>• download films and music: 33.7%<br>• news: 25.1%<br>• chatting: 6.3%<br>• shopping: 5.7%<br>• writing blogs: 5.7%<br>• playing games: 2.9%<br><br>IA correlated with:<br>• Gender (M>F)<br>• Education stage<br>• Time spent of Internet<br>• Games<br>• Communicating with friends and family
Salek Ebrahimi, 2019 [97]	No	General Self-efficacy,	Mean age: 19.73±1.11	IA<br>Normal (<50): 82.9%	No mention of educational work in the paper.	IA Correlations unclear and contradictory.

**Lead Author, Publication Date, Citation**	**Validity of IA questioned?**	**Measured against (e.g. Depression**	**Age & Gender**	**Addiction rates (Prevalence)**	**Mentioned value of Internet for Academic work**	**Results**
		Difficulty in Emotion Regulation, and Resilience	Female: 47.9%<br>Male: 52.1%	At risk (50-79): 15.6%<br>Dependent (80-100): 2.1%		
Samaha, 2019 [98]<br>Samaha, 2018 [99]	No, although acknowledges that there is currently no "gold standard" tool for measurement.	Medical Student Stressor Questionnaire	Mean age: 21.92±2.16<br>Female: 63.4%<br>Male: 36.6%	IA:<br>Normal: 25.2 %<br>Mild: 48.7 %<br>Moderate: 24.8%<br>Severe: 1.3%	No mention in the Introduction.<br>No mention in the paper. One paper is data; the focus was on the internal consistency of the IAT.	IA negatively correlated with:<br>• income<br><br>No correlation on gender
Şenol, 2019 [100]	No	Sagittal alignment (Cobb)	Age range: 18-22<br>Female: 67.3%<br>Male: 32.7%	IA:<br>Not Addicted: 55.4%<br>"Mean addicted": 39.3%<br>Addicted: 5.4%	No, although it does mention quick access to "scientific resources" in general.	Internet used for<br>• "research, lessons, news, music, video and social nets": "about 85%"<br>• games: 7.1%

Lead Author, Publication Date, Citation	Validity of IA questioned?	Measured against (e.g. Depression	Age & Gender	Addiction rates (Prevalence)	Mentioned value of Internet for Academic work	Results
					Does take it into account a little, but still equates usage with addiction.	IA negatively correlated with:<br>• lumbar Cobb angle
Shadzi, 2020 [101]	No	Depression Anxiety Stress Scale (DASS-21); Pittsburgh Sleep Quality Index (PSQI)	Mean age: 22.4±2.18<br><br>Female: 50.3%<br>Male: 49/7%[a]	38.6% had problematic internet use	Not mentioned in Introduction or anywhere else in paper.	Problematic internet use predicted poor sleep quality through indirect pathways by the mediation of mental health problems.
Shi, 2019 [102]	No (in the Discussion, the authors do note that no IA is the gold standard.	Big Five Inventory (BFI); ADHD Self-Report Scale-V1.1;	Mean age: 19.74±1.48<br>Female: 58.9%<br>Male: 41.1%	IA:<br>None: 55.3%[a]<br>Mild: 35.5%<br>Moderate: 8.6%<br>Severe: 0.6%	No mention in paper of value to students.<br><br>Generally high usage levels seen in opposition to work: “They are self-disciplined, diligent and goal striving, so that they can have better control of Internet use, and refrain	IA correlated with:<br>• age group<br>• academic year<br>• hometowns<br>• neuroticism<br><br>IA negatively correlated with:<br>• extraversion<br>• agreeableness<br>• conscientiousness

**Lead Author, Publication Date, Citation**	**Validity of IA questioned?**	**Measured against (e.g. Depression**	**Age & Gender**	**Addiction rates (Prevalence)**	**Mentioned value of Internet for Academic work**	**Results**
					from becoming addicted to it [i.e. the Internet].”	• openness
Shinde, 2018 [103]	No	DASS 21 (Depression)	Mean age cannot be calculated because of conflicting results.<br>Female: 43.33%<br>Male: 56.66%	IA:<br><30: 31.11%<br>30-49: 48.89%<br>50-79: 20%<br>80-100: 0%	Acknowledged in the Introduction.<br><br>No examination of academic or other work; no mention anywhere in the paper.	IA correlated with:<br>• depression
Shoghli, 2018) [6]	No	General health questionnaire	Mean age: 25.29±1.23<br>Female: 51.1%	IA<br>Regular users: 63.5%<br>High risk: 36.5%	Nothing about using the Internet for academic work.	IA correlated with:<br>• marital status (S>M)<br>• maternal occupation<br>• maternal education

Lead Author, Publication Date, Citation	Validity of IA questioned?	Measured against (e.g. Depression	Age & Gender	Addiction rates (Prevalence)	Mentioned value of Internet for Academic work	Results
		(GHQ-28)	Male: 48.9%	Addict: 0%		IA negatively correlated with:<br>• general health
Simcharoen, 2018 [104]	No	Loneliness, interpersonal problems UCLA loneliness scale; Inventory of Interpersonal Problems-32	Mean age: 20.88±1.8<br>Female: 56.8%<br>Male: 43.2%[a]	IA:<br>None: 63.3%<br>Mild: 30.9%<br>Moderate: 5.2%<br>Severe: 0.6%	Acknowledged in the Introduction.<br><br>No mention of academic work elsewhere in the paper.	Internet used for<br>• social networking: 4.52%<br>• chatting 4.46%<br>• YouTube 4.28%<br>• searching for information (e.g. Google) 4.27%<br>• movies/music 3.42%<br>• downloading (movies/music etc.) 3.36%<br>• *reading e-learning 3.08%*<br>• emailing 2.65%<br>• shopping/auction 2.09%<br>• forum participation (Web board): 1.96%<br>• gaming 1.92%<br>• banking/business: 1.56%<br>• blogging/diary: 1.22%<br>• gambling 1.04%
Singh, 2018 [105]	No (although	Epworth Sleepine	Mean age: 21.9±4.6 years	IA<br>Normal: 34.23%<br>Mild: 57.72%	No mention in the Introduction.	IA correlated with:<br>• excessive daytime sleepiness

**Lead Author, Publication Date, Citation**	**Validity of IA questioned?**	**Measured against (e.g. Depression**	**Age & Gender**	**Addiction rates (Prevalence)**	**Mentioned value of Internet for Academic work**	**Results**
	acknowledges that it is not in DSM)	ss Scale (ESS)	Females: 71.82%<br>Males: 28.18%	Moderate: 7.38%<br>Severe: 0.67%	No mention of academic work in the paper.	
Singh, 2018 [106]	No, but does acknowledge that "Measurement of level of problematic internet use has been a challenge"	Problematic Internet behaviours.	Mean age: 20.6±0.88<br>Female: 21.3%[a]<br>Males: 78.7%	IA:<br>Average: 80.3%<br>Moderate: 19.7%<br>Severe: 0%	No mention of work. But they state this:<br>Find higher Internet usage in the classroom among those with higher IA scores, but do not consider the possibility that this usage may be work-related.	IA correlated with:<br>• use of email<br>• social networking<br>• blogging<br>• forums<br>• online leisure activities<br>• surfing without purpose<br>• online shopping<br>• downloading
Siraj, 2015 [107]	No	cGPA	Female: 73%<br>Males: 27%	Internet user > 6 hours were observed to have higher CGPA	Discussed in some detail in Introduction.<br><br>Quite a bit of discussion on the topic. Does not explicitly modify the interpretation of the	Internet used for:<br>• *course-related: 84.1 %*<br>• entertainment: 76.1%<br>• *assignment: 71.6%*<br>• non-course related: 43.2%<br>• chat: 38.1%<br>• email: 35.8%

Lead Author, Publication Date, Citation	Validity of IA questioned?	Measured against (e.g. Depression	Age & Gender	Addiction rates (Prevalence)	Mentioned value of Internet for Academic work	Results
					addiction figures, but does somewhat put it into perspective.	• music: 33.5%<br>• play game: 11.4%<br>• download game: 4.5%<br>• create web page: 1.7%
Srijampana, 2014 [108]	No, although does acknowledge that there is a debate about how best to classify it.	None	Mean age: 19.9<br>Female: 57.2%<br>Male: 42.8%	Less than average: 23.2%<br>Average users: 64.4%<br>Possible: 11.8%,<br>Addicts: 0.4%	Acknowledged in Introduction.<br><br>Incomplete/contradictory data on academic usage.	Internet used for:<br>• social networking: 59.7%<br>• downloading media files: 18.9%<br>• online gaming: 12.3%<br>• *academic purposes: 9.0%*<br><br>Correlation only of social media sites with gender (M>F).
Subhaprada, 2017 [109]	No	Academic performance (self-reported, no details)	Females: 37.8%<br>Males: 62.2%	IA:<br>None: 23.16%<br>Mild: 52.63%<br>Moderate: 24.21%<br>Severe: 0%	Acknowledged in the Introduction, no mention in discussion	IA correlated with:<br>• gender (M>F)<br><br>IA negatively correlated with:<br>• academic performance.
Suresh, 2018 [110]	Yes, on definitio	Lyubomirsky		IAT:<br>None: 12.6%	Acknowledged in the Introduction.	Higher levels of internet addiction showed lower

Lead Author, Publication Date, Citation	Validity of IA questioned?	Measured against (e.g. Depression	Age & Gender	Addiction rates (Prevalence)	Mentioned value of Internet for Academic work	Results
	n and whether it should be included in DSM.	and Lepper's Subjective Happiness Scale		Mild: 58.2%<br>Moderate: 28%<br>Severe: 0.7%[a]	No mention in Discussion, although the Conclusion does point out that the Internet is important for academic work. This, however, does not influence the interpretation of the results.	levels of subjective happiness
Sushma, 2018 [111]	No	None	Mean age: 20.6±1.97<br><br>Female: 37.29%<br>Male: 62.17%	IA:<br>None: 21.2%<br>Mild: 58.2%<br>Moderate: 19.5%<br>Severe: 0.8%	Not mentioned in Introduction<br><br>No mention in the Discussion.	Internet used for:<br>• social networking: 25%<br>• media: 24.20%<br>• *academics: 15.3%*<br>• games: 10.2%<br>• others: 25.4%<br><br>IA correlated with:<br>• gender (M>F)<br>• time spent on using internet per day
Taha, 2019 [11]	No Incorrectly states that it is included	None	Females: 42.1%[a]<br>Males: 57.9%	IA:<br>Lower than average: 2.9%<br>Average: 26.8%	Not mentioned in Introduction.<br><br>In limitations, does acknowledge that "some	IA correlated with:<br>• gender (F>M)<br>• year of study: 2nd year > 1st and 3rd year

Lead Author, Publication Date, Citation	Validity of IA questioned?	Measured against (e.g. Depression	Age & Gender	Addiction rates (Prevalence)	Mentioned value of Internet for Academic work	Results
	in the DSM.			Possible addict: 57.9%<br>Addict: 12.4%	of the students may have been using the Internet for work- or research-related purposes", but this does not ever affect the interpretation of their results as "addiction"	• neck pain<br>• sleeplessness<br><br>Problems also reported: headaches, backache, weight gain, neck pain and other psychological problems
Tan, 2019 [7]	No, acknowledges that it is not in the DSM, but suggests that it should be.	UCLA Loneliness Scale; Academic Expectations Stress Inventory; Multidimensional Scale of Perceived Social Support	Mean age: 21.01±1.09<br>Female: 70%<br>Male: 30%	IA: 31.9%	Not mentioned in the paper.	IA negatively correlated with:<br>• social support from family<br>• social support from friends<br>• social support from significant other

**Lead Author, Publication Date, Citation**	**Validity of IA questioned?**	**Measured against (e.g. Depression**	**Age & Gender**	**Addiction rates (Prevalence)**	**Mentioned value of Internet for Academic work**	**Results**
Tsimtsiou, 2014 [112]	No	None	Mean age: 21.1±3.5	Mean IAT score: 31.2 (SD = 16.4)<br>Normal: 52.9 %<br>Mild: 31.4 %<br>Moderate: 15.7 %	No discussion of Internet for work purposes anywhere in the paper.	Internet used for:<br>• gambling: 39.1%<br>• gaming: 35.1%<br>• adult entertainment: 35.1%<br><br>IA correlated with:<br>• gambling<br>• pornographic sites<br>• games
Tsimtsiou, 2015 [113]	No	None	Mean age: 21.34[a]	IA:<br>IA Mean score: 25.4 (SD=13.9)<br>Normal: 69.9%<br>Mild: 24.5%<br>Moderate: 5.4%<br>Severe: 0.2%	Not mentioned anywhere in the paper; even the fact that 76% of the students use the Internet for their education is ignored in the Discussion.	Internet used for:[a]<br>• Facebook: 80.15%<br>• e-mail: 77.90%<br>• *education: 75.84%*<br><br>IA correlated with:<br>• visits in Internet cafes<br>• *at school*<br>• via mobile application<br>• Facebook,<br>• Twitter<br>• online games<br>• mean duration of Internet use

Lead Author, Publication Date, Citation	Validity of IA questioned?	Measured against (e.g. Depression	Age & Gender	Addiction rates (Prevalence)	Mentioned value of Internet for Academic work	Results
Upadhayay, 2017 [114]	No	None	Females: 50%<br>Males: 50%	IA:<br>None: 79%<br>"Slightly Addicted": 21%	Not mentioned in Introduction.<br><br>Use of Internet for education acknowledged in the Discussion, although makes the point that none of those who said it was a priority falls into the Addicted group.	Highest priority to using the Internet:<br>• movies/songs: 18%<br>• *educational and learning activities: 17%*<br>• communicating with friends and families: 14%
V, 2017 [115]	No	Depression, anxiety and stress (DASS 42).	No age or gender data given.	IA[a]:<br>None: 29.7%<br>Mild: 49.1%<br>Moderate: 19.1%<br>Severe: 2.0%	No mention made of work anywhere in the paper.	IA correlated with:<br>• anxiety levels
Vidyachathoth, 2013 [116]	Does acknowledge that there was some debate prior to DSM-5	Affect (PANAS)	Mean age: 18.49±0.71<br><br>Female: 62.2%<br>Males: 37.8%		No mention in the Introduction.<br><br>Only leisure work studied, so no mention of work.	IA negatively correlated with:<br>• Affects score.

Lead Author, Publication Date, Citation	Validity of IA questioned?	Measured against (e.g. Depression	Age & Gender	Addiction rates (Prevalence)	Mentioned value of Internet for Academic work	Results
Wang, 2020 [117]	No	Pittsburgh Sleep Quality Index (PSQI); Self-Harm Questionnaire	Mean age: 18.8[a]<br>Female: 58.48%<br>Male: 41.52%	28.2% reported having IA (IA score > 40).	Not mentioned in Introduction.<br><br>Use of the Internet for work ignored.	IA correlated with:<br>• gender (F>M)<br>• upper grade students<br>• poor sleep quality<br>• possibility of self-injury
Yerpude, 2019 [118]	No	Mental health inventory	Mean age: 19.32±1.64<br><br>Female: 58.33%<br>Male: 41.67%	Prevalence: 23.48%	Acknowledged in the Introduction.<br><br>No mention of work-related activities in the paper.	Internet used for:<br>• social networking: 35.61%<br>• *educational: 25.76%*<br>• recreational: 21.21%<br>• games: 17.42%<br><br>IA correlated with:<br>• gender (M>F)<br>• computer ownership<br>• login status<br>• mode of internet access<br>• anxiety<br>• depression

Lead Author, Publication Date, Citation	Validity of IA questioned?	Measured against (e.g. Depression	Age & Gender	Addiction rates (Prevalence)	Mentioned value of Internet for Academic work	Results
						• loss of emotional/behavioral control<br>• emotional ties<br>• psychological distress
Yücens, 2018 [119]	Yes; indicates that it is not in the DSM, and other indications that definition is difficult. Also, that IA may be some form of self-medication	Liebowitz Social Anxiety Scale (LSAS); Barratt Impulsivity Scale-11 (BIS-11) Rosenberg Self-Esteem Scale (RSES); Beck Depression Inventory (BDI);	Female: 57.4%[a]<br>Male: 42.6%[a]	IA<br>None (<50): 73%<br>Moderate: 23.7%<br>Severe: 3.3%	Acknowledged in Introduction<br><br>Not mentioned anywhere in the paper.<br><br>In spite of the caveats in the Introduction, this does not appear to affect the interpretation of addiction rates.	IA correlated with:<br>• anxiety (including social anxiety)<br>• depression<br><br>IA negatively correlated with:<br>• self-esteem

**Lead Author, Publication Date, Citation**	**Validity of IA questioned?**	**Measured against (e.g. Depression**	**Age & Gender**	**Addiction rates (Prevalence)**	**Mentioned value of Internet for Academic work**	**Results**
	against other problems. It may resemble ICD more than an addiction	Beck Anxiety Inventory (BAI).				

[a] Not stated in the paper, but calculated based upon the other percentages and raw data.
[b] The authors did not use the standard classification; instead, they used None (<40), Possible (40-69), Addicted:>70
